# Proteomic and phosphoproteomic analyses of myectomy tissue reveals difference between sarcomeric and genotype-negative hypertrophic cardiomyopathy

**DOI:** 10.1038/s41598-023-40795-1

**Published:** 2023-09-01

**Authors:** Ramin Garmany, J. Martijn Bos, Surendra Dasari, Kenneth L. Johnson, David J. Tester, John R. Giudicessi, Cristobal dos Remedios, Joseph J. Maleszewski, Steve R. Ommen, Joseph A. Dearani, Michael J. Ackerman

**Affiliations:** 1 Mayo Clinic Graduate School of Biomedical Sciences, Mayo Clinic Alix School of Medicine and the Mayo Clinic Medical Scientist Training Program, Rochester, MN USA; 2 Department of Molecular Pharmacology & Experimental Therapeutics, Windland Smith Rice Sudden Death Genomics Laboratory, Mayo Clinic, Rochester, MN USA; 3 Department of Cardiovascular Medicine, Windland Smith Rice Genetic Heart Rhythm Clinic, Mayo Clinic, Rochester, MN USA; 4 Department of Pediatric and Adolescent Medicine/Division of Pediatric Cardiology, Mayo Clinic, Rochester, MN USA; 5 Department of Quantitative Health Sciences/Division of Computational Biology, Mayo Clinic, Rochester, MN USA; 6 Proteomics Core, Mayo Clinic, Rochester, MN USA; 7 Mechanobiology Laboratory, Victor Chang Cardiac Research Institute, Darlinghurst, Australia; 8 Department of Laboratory Medicine and Pathology, Mayo Clinic, Rochester, MN USA; 9 Department of Cardiovascular Surgery, Mayo Clinic, Rochester, MN USA; 10 Mayo Clinic Windland Smith Rice Genetic Heart Rhythm Clinic and Windland Smith Rice Sudden Death Genomics Laboratory, Mayo Clinic, Guggenheim 501, 200 First Street SW, Rochester, MN 55905 USA

**Keywords:** Proteome informatics, Cardiology, Functional genomics, Genotype, Medical genetics

## Abstract

Hypertrophic cardiomyopathy (HCM) is a genetically heterogenous condition with about half of cases remaining genetically elusive or non-genetic in origin. HCM patients with a positive genetic test (HCM_Sarc_) present earlier and with more severe disease than those with a negative genetic test (HCM_Neg_). We hypothesized these differences may be due to and/or reflect proteomic and phosphoproteomic differences between the two groups. TMT-labeled mass spectrometry was performed on 15 HCM_Sarc_, 8 HCM_Neg_, and 7 control samples. There were 243 proteins differentially expressed and 257 proteins differentially phosphorylated between HCM_Sarc_ and HCM_Neg_. About 90% of pathways altered between genotypes were in disease-related pathways and HCM_Sarc_ showed enhanced proteomic and phosphoproteomic alterations in these pathways. Thus, we show HCM_Sarc_ has enhanced proteomic and phosphoproteomic dysregulation observed which may contribute to the more severe disease phenotype.

## Introduction

Hypertrophic cardiomyopathy (HCM) is one of the most common genetic heart diseases and is associated with pathogenic genetic variants in genes that encode sarcomeric proteins. To date, at least 27 HCM-susceptibility genes have been discovered^1^. However, about half of patients remain genetically elusive with no identified HCM-associated genetic variant^2,3^. Importantly, there are clear phenotypic differences between sarcomere-positive HCM (HCM_Sarc_) and genotype-negative HCM (HCM_Neg_). Patients with HCM_Sarc_ have a more severe disease phenotype, more severe disease progression, and worse outcomes^4–7^. Recent proteomic analysis of HCM have uncovered a wide network of pathways altered in HCM likely responsible for disease development^7–10^. Additionally, we recently demonstrated that HCM is characterized by widespread alterations in the proteome^7^. Despite clear differences between HCM and healthy individuals, the overall proteomic architecture of HCM was similar irrespective of genotype. However, given the clinical differences between genotypes, we hypothesized there may be subtle differences in the (phospho)proteome when comparing the (phospho)proteome between those with a positive genetic test (HCM_Sarc_) versus those with a negative genetic test (HCM_Neg_).

## Methods

We performed a subgroups analysis using our previously published proteomics cohort^7^. All patients provided written informed consent for this Mayo Clinic Institutional Review Board (IRB 811-98) approved study which abides by the Declaration of Helsinki. Detailed methods are provided in Supplemental Data. Briefly, patients with diagnosed obstructive HCM who underwent clinically indicated surgical myectomy for the relief of outflow tract obstruction were genotyped by genome sequencing followed by variant adjudication using American College of Medical Genetics and Genomics (ACMG) criteria^11^ and divided into genotype subgroups: HCM_Sarc_ (N = 15) defined as patients having an ACMG graded pathogenic (P)/likely pathogenic (LP) variant in a definitive or strong evidence HCM-susceptibility gene encoding a sarcomeric protein, and HCM_Neg_ (N = 8) defined as patients with no variants of any classification in a panel of 54 HCM-associated genes^1,7^. Any patient with variants in HCM mimicker genes were excluded^1^. Genome sequencing was used to confirm the HCM_Neg_ did not have deep intronic variants. Additionally, control tissue samples (N = 7) from normal donor hearts for which a suitable heart transplant recipient was not identified, were included in this study. All samples underwent TMT-labeled mass-spectrometry for proteomic and phosphoproteomic analysis. Differential expression analysis was performed to identify differences between subgroups followed by pre-ranked gene set enrichment analysis (GSEA) to identify which Gene Ontology (GO) biological processes were enriched in the proteome and phosphoproteome of HCM_Sarc_ compared to HCM_Neg_. Finally, differentially expressed (phospho)proteins were inputted into Ingenuity Pathway Analysis (IPA) for identifying altered pathways.

## Results

### Direct comparison of HCM_Sarc_ and HCM_Neg_

HCM_Sarc_ patients were diagnosed at a younger age (33 ± 16 vs. 47 ± 19 years; p = 0.04) and had their myectomies done at a younger age (40 ± 17 vs. 52 ± 16 years; p = 0.02) compared with HCM_Neg_ patients (Supplemental Table 1). To test the hypothesis that HCM_Sarc_ and HCM_Neg_ have alterations in pathways that may be responsible for observed clinical differences, we directly compared the proteomes and phosphoproteomes of HCM_Sarc_ and HCM_Neg_ myectomy samples. Consistent with previous studies^7,8^, the proteome of HCM was similar regardless of genotype with no clear separation on principal component analysis (PCA) plotting (Supplemental Fig. S1A). Nonetheless, a direct comparison of the proteome of HCM_Sarc_ with HCM_Neg_ revealed 243 differentially expressed proteins (DEPs) with 102 up-regulated and 141 down-regulated proteins (Supplemental Fig. S1B).

Overall, 4213 phosphorylated proteins were detected across all samples with PCA analysis showing slight, but not entirely complete separation between HCM_Sarc_ and HCM_Neg_ (Supplemental Fig. S1C). Differential analysis identified 257 differentially phosphorylated proteins (DPPs): 134 hypophosphorylated and 123 hyperphosphorylated (Supplemental Fig. S1D). A complete list of DPPs can be found in the Supplemental Data.

**Figure 1:**
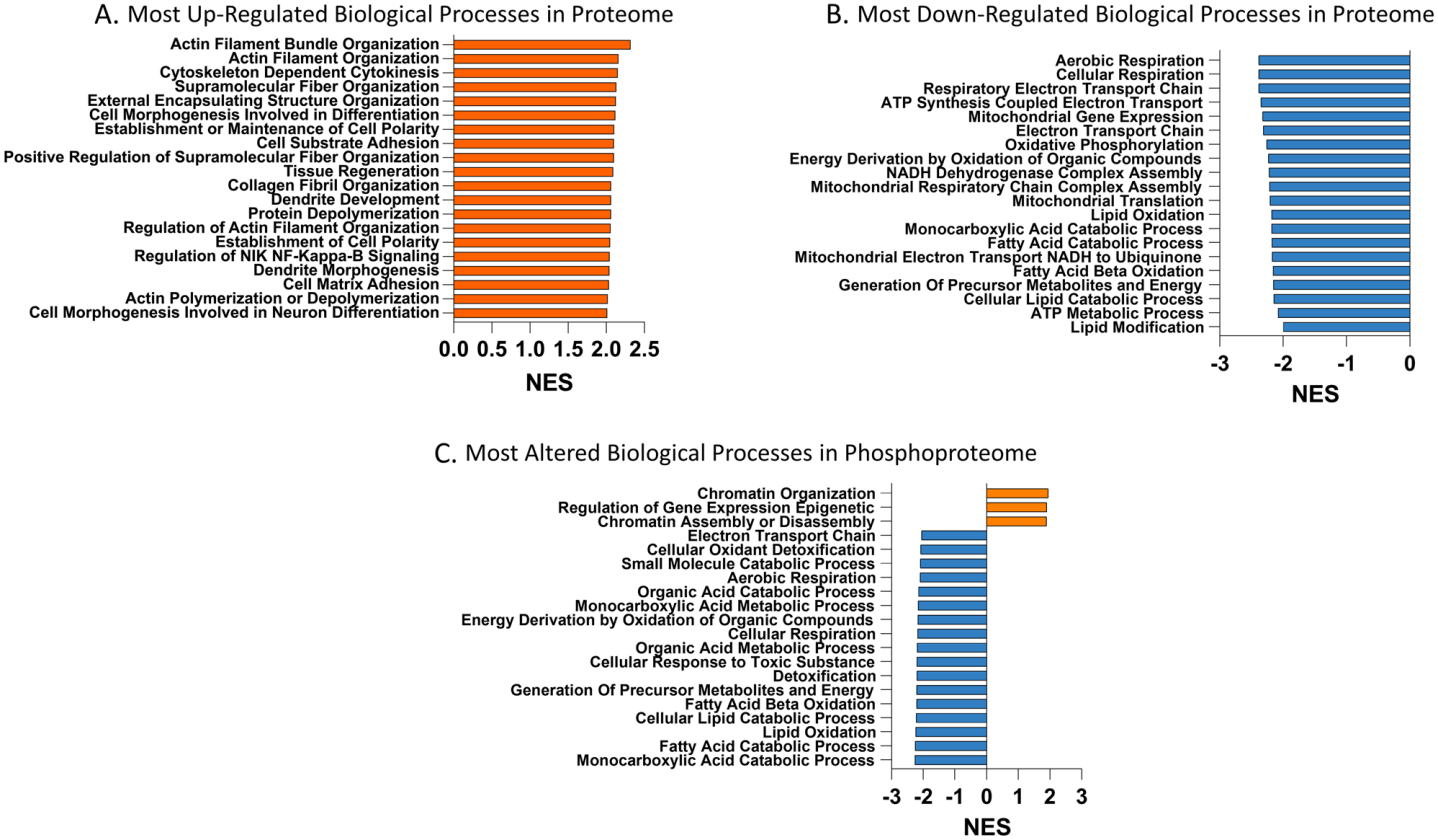
Comparison of gene ontology (GO) biological processes altered between HCM_Sarc_ and HCM_Neg_. (**A**) Most up-regulated gene ontology (GO) biological processes in the proteome using gene set enrichment analysis (GSEA). (**B**) Most down-regulated GO biological processes in proteome using GSEA. (**C**) Most altered GO biological processes in phosphoproteome using GSEA.

Next pre-ranked, GSEA identified 128 biological processes altered at the proteome level with 97 being up-regulated and 31 down-regulated in HCM_Sarc_ compared with HCM_Neg_ (Supplement). The top up-regulated processes were involved in either cell adhesion, extracellular matrix formation, and activation of fibrosis or regulation of cytoskeletal processes (Fig. 1A). In contrast, the top down-regulated processes were entirely metabolic processes with predominant down-regulation of aerobic respiration and mitochondrial function and catabolic processes, especially of fatty acids (Fig. 1B). At the phosphoproteome level, 57 biological processes were altered with 3 up-regulated and 54 down-regulated (Fig. 1C) with the main up-regulated processes involved in chromatin organization and gene expression while the majority of down-regulated processes were metabolic pathways akin to what was observed in the proteome.

**Figure 2:**
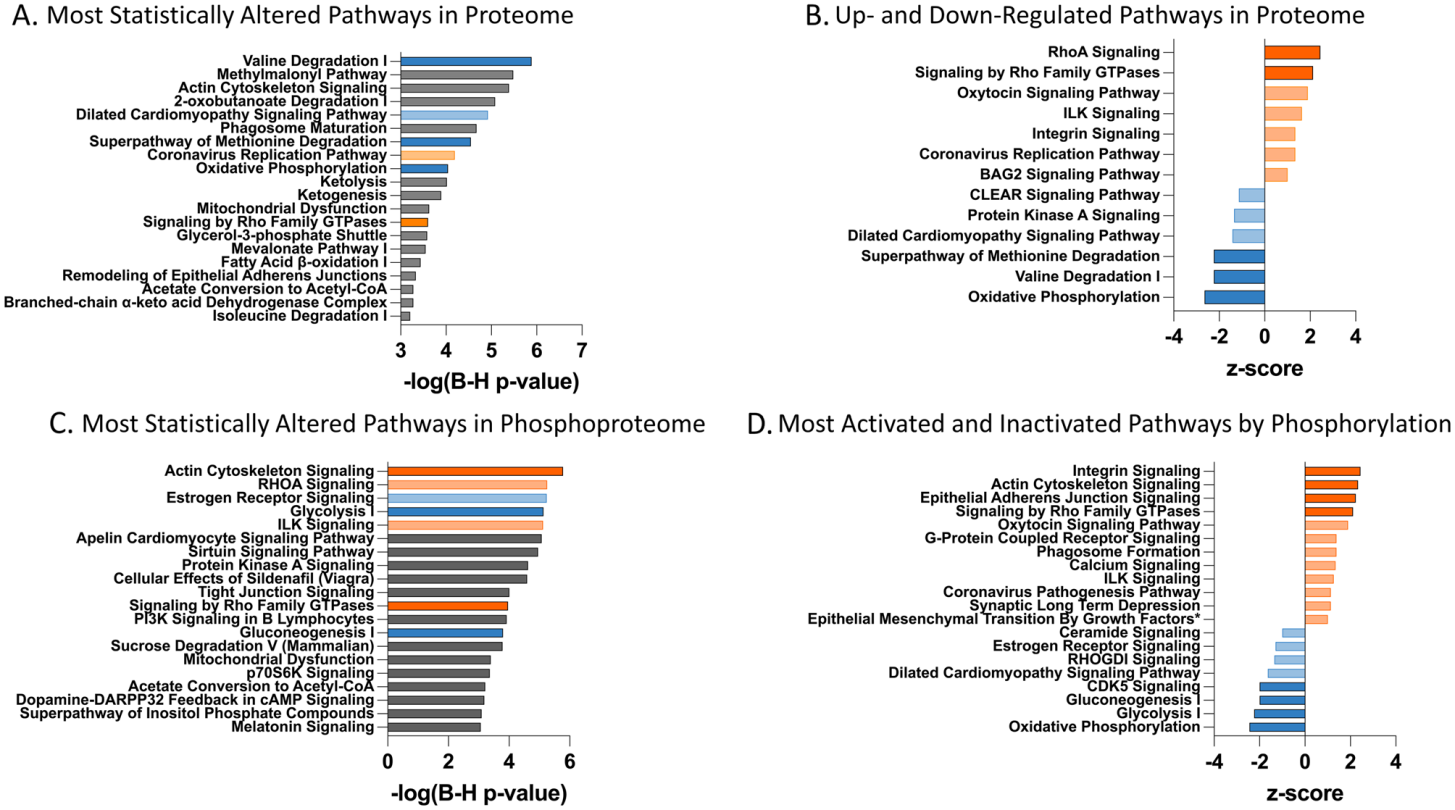
Comparison of pathways altered between HCM_Sarc_ and HCM_Neg_. (**A**) Most statistically altered pathways (largest-log [BH p-value]) in proteome. (**B**) Top up- and down-regulated pathways in proteome. (**C**) Most statistically altered phosphorylation (largest-log [BH p-value]) of pathways. (**D**) Top activated and inactivated pathways based on phosphorylation (z-score ≥|1|). *Regulation of the epithelial mesenchymal transition by growth factors pathway was shortened.

Finally, DEPs and DPPs were inputted into Ingenuity Pathway Analysis (IPA) to identify alterations in canonical pathways. Overall, 72 pathways were altered at the proteome level with 7 up-regulated (z-score ≥ 1), 6 down-regulated (z-score ≤ -1), and 59 pathways for which directionality was indeterminate (Supplement). The valine degradation I, methylmalonyl pathway, actin cytoskeletal signaling, 2-oxobutanoate degradation I, and dilated cardiomyopathy signaling pathways were the most statistically altered pathways (largest -log [BH p-value]) (Fig. 2A). RhoA signaling (z-score = 2.5) and signaling by Rho Family GTPases (z-score = 2.1) were the two most up-regulated pathways in HCM_Sarc_. In addition, oxytocin signaling pathway (z-score = 1.89), integrin-linked kinase (ILK) pathway (z-score = 1.6), integrin signaling (z-score = 1.3), coronavirus replication pathway (z-score = 1.3), and BAG2 signaling pathway (z-score = 1.0) were moderately up-regulated (Fig. 2B).

In addition, ketolysis, ketogenesis, mitochondrial dysfunction, glycerol-3-phosphate shuttle, fatty acid β-oxidation, acetate conversion to acetyl-CoA, branched-chain α-keto acid dehydrogenase complex were altered significantly. Oxidative phosphorylation was the most down-regulated pathway (z-score = − 2.7; Fig. 2B). Collectively, HCM_Sarc_ showed widespread down-regulation of aerobic respiration, mitochondrial function, and catabolic pathways compared with HCM_Neg_.

Pathway analysis of the DPPs demonstrated that 109 pathways had significant alterations in protein phosphorylation with 14 pathways predicted to be activated (z-score ≥ 1) and 8 inactivated (z-score ≤ − 1); for 87 pathways the directionality was unclear (Supplement). The pathways with the most alteration in phosphorylation status (largest − log [BH p-value]) are presented in Fig. 2C and included actin cytoskeleton signaling, RhoA signaling, estrogen receptor signaling, glycolysis I, ILK signaling, and signaling by Rho family GTPases. The pathways predicted to be activated or inactivated due to changes in phosphorylation are shown in Fig. 2D with activation of integrin signaling, actin cytoskeleton signaling, epithelial adherens junction signaling, signaling by Rho family GTPases, oxytocin signaling pathway, G-protein coupled receptor signaling, phagosome formation, calcium signaling, ILK signaling, coronavirus pathogenesis pathway, synaptic long-term depression, and epithelial mesenchymal transition by growth factors**.** Many metabolic pathways, including oxidative phosphorylation, glycolysis, gluconeogenesis, and dilated cardiomyopathy signaling pathways were predicted to be inactivated based on changes in phosphorylation (Fig. 2D). Overall, these results show significant concordance between proteomic and phosphoproteomic alterations supporting distinct differential regulation between HCM_Sarc_ and HCM_Neg_.

**Figure 3:**
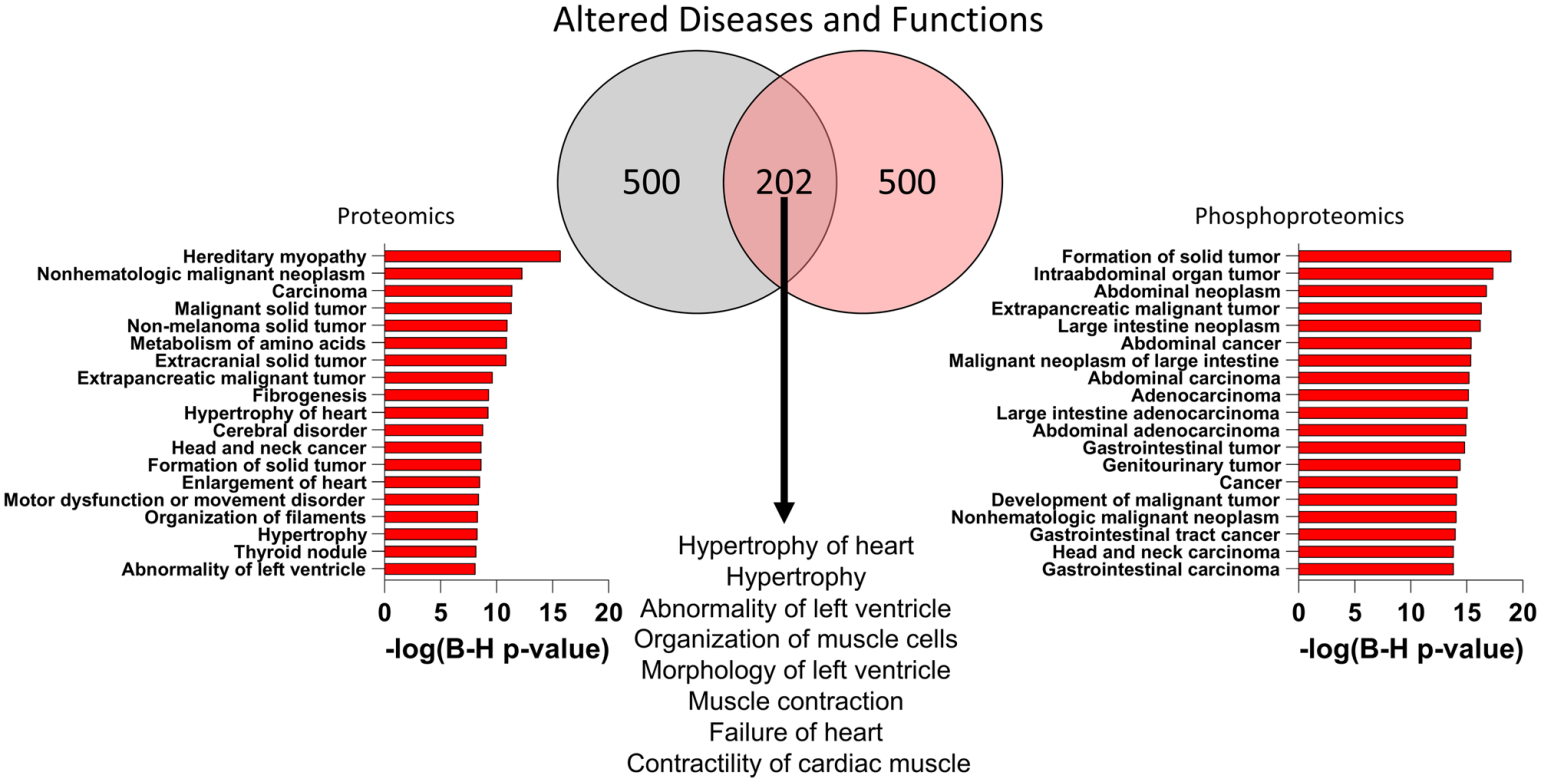
Altered diseases and functions in proteome and phosphoproteome identified using ingenuity pathway analysis.

Additionally, the DEPs and DPPs were analyzed to identify enrichment for altered diseases and processes with the top 20 most statistically altered shown in Fig. 3. Of note, the disease processes altered at the proteome level were relevant to HCM such as hereditary myopathy, fibrogenesis, hypertrophy of heart, enlargement of heart. While growth and cancer processes were altered in both the proteome and phosphoproteome, they were more prominent at the phosphoproteome level. Thus, the proteomic and phosphoproteomic differences are predicted to impact cardiac function and cardiac hypertrophy demonstrating the changes between these genotypes are in disease relevant pathways.

### Comparisons of HCM with controls

In addition to HCM_Sarc_ and HCM_Neg_ comparisons, we subsequently compared the phospho(proteomes) of HCM_Sarc_ and HCM_Neg_ to non-hypertrophied normal cardiac tissue (controls) to identify disease-specific pathways. As expected, there was prominent dysregulation in the proteome and phosphoproteome of both HCM_Sarc_ and HCM_Neg_ compared with controls. Overall, HCM was characterized by up-regulation of cytoskeletal and hypertrophy pathways and down-regulation of metabolic pathways. These findings are summarized in the Supplemental Results and Supplemental Figs. S2–S5.

### Commonalities and differences in the proteome and phosphoproteome of HCM_Sarc_ and HCM_Neg_

**Figure 4:**
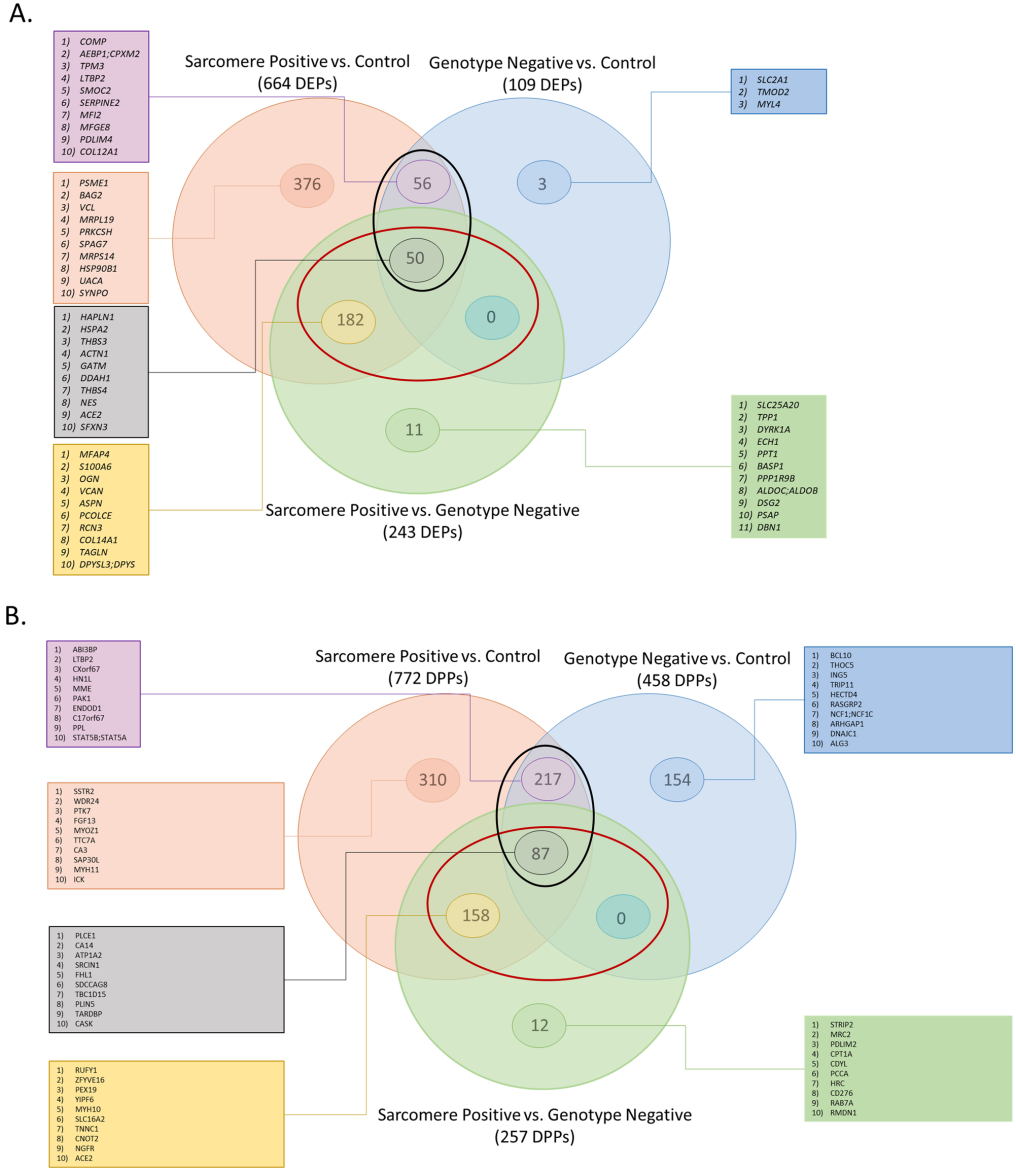

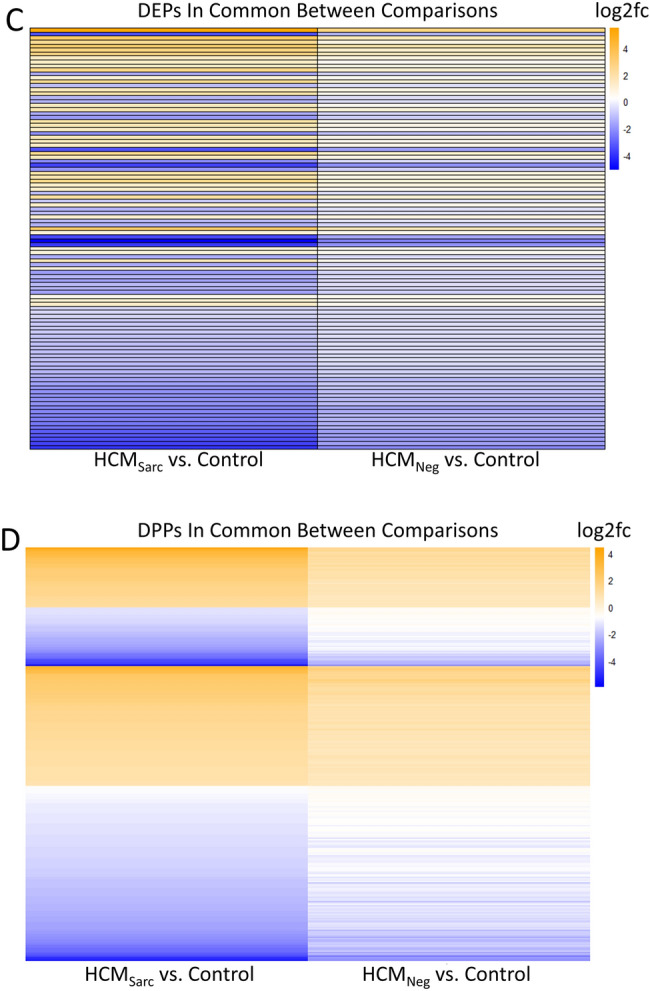
Commonalities and differences in the proteome and phosphoproteome of HCM_Sarc_ and HCM_Neg_. (**A**) Venn diagram comparing differentially expressed proteins (DEPs) between different comparisons. (**B**) Venn diagram comparing differentially phosphorylated proteins (DPPs) between different comparisons. (**C**) Heatmap showing directionality of differentially expressed proteins (DEPs) altered in both HCM_Sarc_ and HCM_Neg_ compared with controls. (**D**) Heatmap showing directionality of differentially phosphorylated proteins (DPPs) altered in both HCM_Sarc_ and HCM_Neg_ compared with controls. Log2fc, log2 fold change.

Next, the three comparisons: HCM_Sarc_ versus HCM_Neg_, HCM_Sarc_ versus controls, and HCM_Neg_ versus controls were juxtaposed to identify genotype-specific differences and similarities. Venn diagrams were generated to summarize the findings. Changes found in the HCM_Sarc_ versus controls and HCM_Neg_ versus control comparisons are considered disease-relevant changes. Overall, there was significant overlap between the proteins and phosphoproteins altered in HCM_Sarc_ versus HCM_Neg_ and those altered in disease (Fig. 4A,B; red circle). In fact, 232/243 (95%) DEPs in HCM_Sarc_ versus HCM_Neg_ were also altered when comparing disease to controls (Fig. 4A). Integrating the DPPs across the three phosphoproteome comparisons revealed most of the direct differences between HCM_Sarc_ and HCM_Neg_ (245/257 [95%]) were in phosphoproteins also observed to be altered in disease compared with controls (Fig. 4B; red circle). Thus, both at the protein and phosphoprotein level, most of the changes between subtypes were in disease-altered proteins (proteins altered in HCM compared to controls).

Overall, there were 376/664 (57%) DEPs found in HCM_Sarc_ compared with controls that were not seen when comparing to HCM_Neg_ (Fig. 4A; orange circle). These HCM_Sarc_ specific DEPs were used to generate a protein–protein interaction network followed by enrichment analysis for biological processes which demonstrated predominant enrichment for metabolic gene sets (Supplemental Fig. 6). Since there were only 3 DEPs unique to HCM_Neg_ versus controls, no genotype specific network was generated (Fig. 4A; blue circle).

There were 106 DEPs found when comparing both HCM_Sarc_ versus controls and HCM_Neg_ versus controls (Fig. 4A; black circle). Figure 4C shows that the direction of change for all the DEPs is the same between both comparisons. There were 304 DPPs altered in both HCM_Sarc_ versus controls and HCM_Neg_ versus controls (Fig. 4B; black circle). Figure 4D shows that the direction of change for all the DPPs is the same between both comparisons.

Considering GO biological processes, there was a similar, prominent overlap across the different genotype comparisons (Supplemental Fig. S7A). Since GSEA uses the entire protein list and not only differentially expressed proteins, more differences were observed. Still, 84/128 (66%) biological processes altered in HCM_Sarc_ versus HCM_Neg_ were also altered in disease versus controls (red circle). There were 44/128 (34%) biological processes altered specifically in HCM_Sarc_ compared with HCM_Neg_ (green circle). Notably, across the comparisons, the biological processes were similar throughout and were mostly extracellular matrix and cytoskeletal processes or metabolic processes. Interestingly, GO biological processes altered due to phosphorylation only had 24/57 (42%) of those altered in HCM_Sarc_ compared with HCM_Neg_ also altered in disease compared with controls (Supplemental Fig. S7B; yellow circle). Specifically, there were epigenetic processes (i.e., chromatin organization) processes along with metabolic processes altered uniquely between the genotypes but not in disease compared with controls.

**Figure 5:**
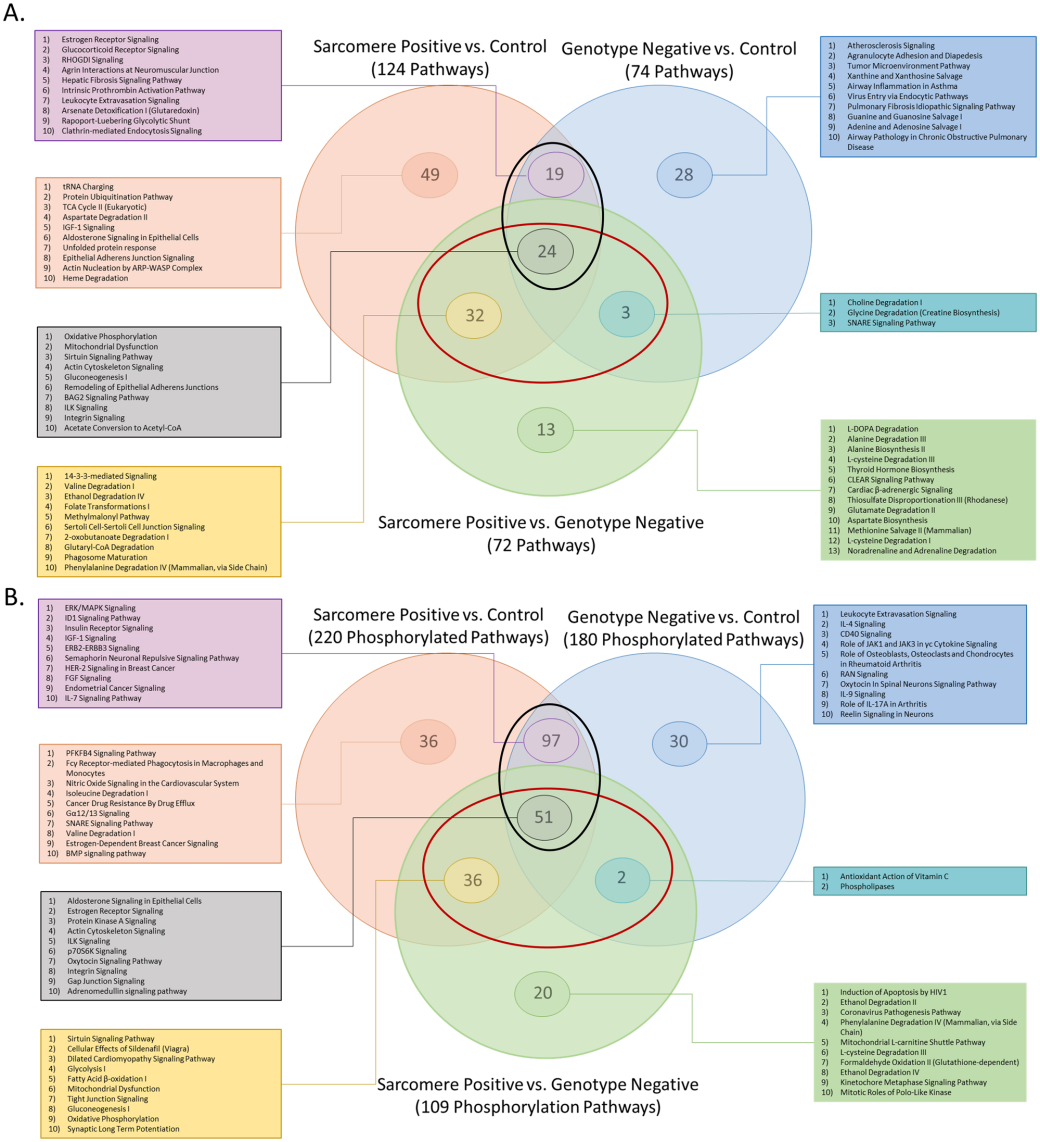

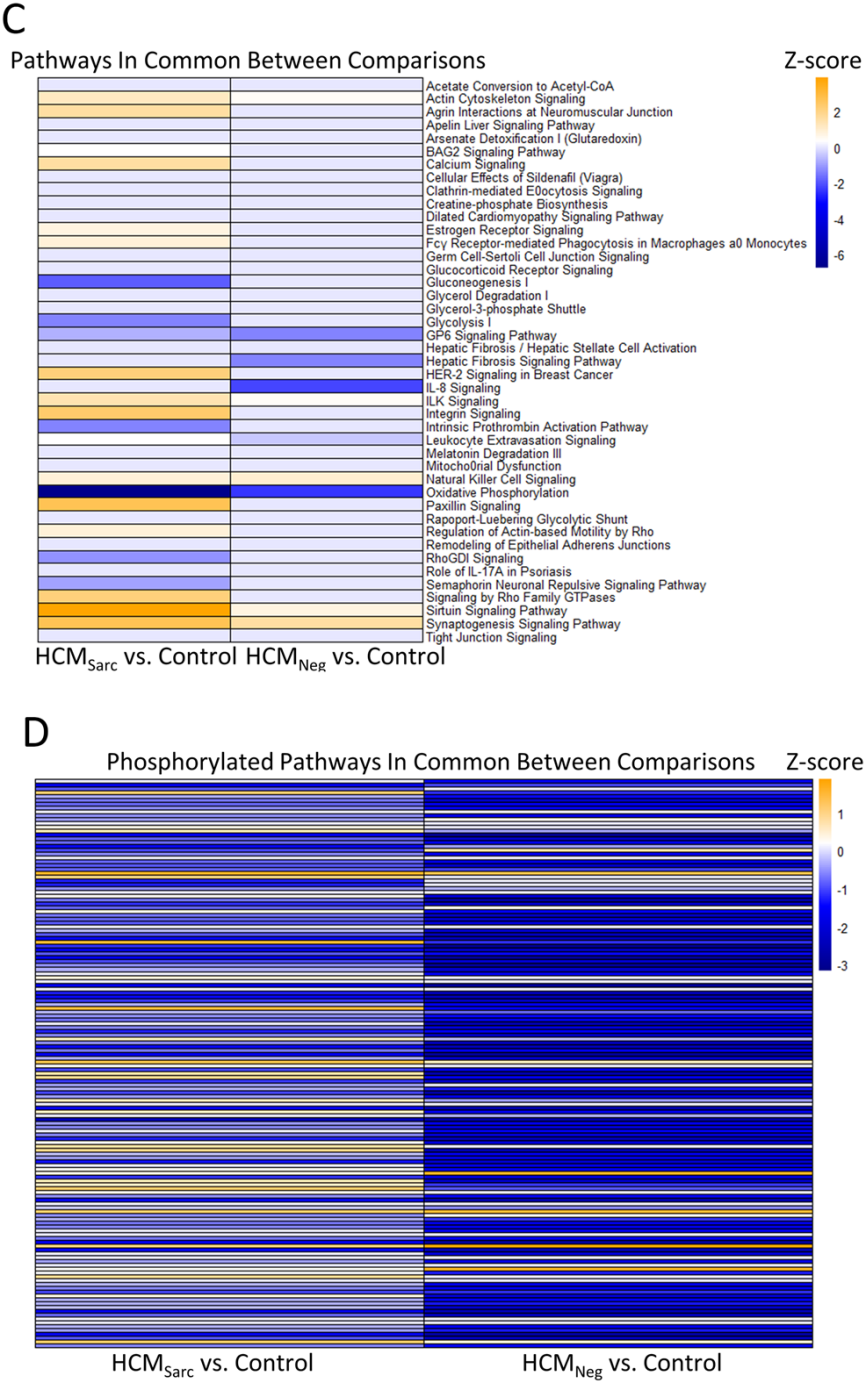
Commonalities and differences in the proteome and phosphoproteome of HCM_Sarc_ and HCM_Neg_ continued. (**A**) Venn diagram comparing pathways altered in proteome between different comparisons. (**B**) Venn diagram comparing pathways with altered phosphorylation between different comparisons. (**C**) Heatmap showing directionality of pathways altered in both HCM_Sarc_ and HCM_Neg_ compared with controls. (**D**) Heatmap showing directionality of phosphorylated pathways altered in both HCM_Sarc_ and HCM_Neg_ compared with controls.

At the pathway level, the overlap between the genotype comparisons was very clear with 59/72 (82%) pathways altered between HCM_Sarc_ and HCM_Neg_ also altered between disease and controls (Fig. 5A; red circle). There were 43 pathways altered at the proteome level both when comparing HCM_Sarc_ with controls and HCM_Neg_ with controls suggesting these pathways likely serve a central role in maladaptive cardiac hypertrophy regardless of genetic background (black circle).Twenty-four pathways were common to all proteomic comparisons including HCM_Sarc_ versus HCM_Neg_ such as oxidative phosphorylation, mitochondrial dysfunction, sirtuin signaling pathway, actin cytoskeleton, gluconeogenesis I, remodeling of epithelial adherens junctions, ILK signaling, integrin signaling, dilated cardiomyopathy pathway, signaling by Rho Family GTPases, and calcium signaling (Fig. 5A; gray circle and Supplement).

There were even greater numbers of pathways impacted due to phosphorylation with 89/109 (82%) pathways altered in both HCM_Sarc_ compared with HCM_Neg_ and disease compared with controls (Fig. 5B; red circle). Fifty-one pathways were altered due to phosphorylation in all comparisons including aldosterone signaling in epithelial cells, estrogen receptor signaling, protein kinase A signaling, and actin cytoskeleton signaling (Fig. 5B; gray circle and Supplement).

There were 43 pathways altered when comparing both HCM_Sarc_ and HCM_Neg_ compared with controls (Fig. 5A; black circle). Of these 43 pathways the directionality of all changes were concordant between both comparisons (Fig. 5C; Supplemental Table S2). These included pathways such as actin cytoskeleton signaling, BAG2 signaling, calcium signaling, dilated cardiomyopathy signaling, gluconeogenesis, glycolysis, ILK signaling, integrin signaling, oxidative phosphorylation, RhoGDI signaling, and signaling by Rho Family GTPases. There were 148 phosphorylated pathways common between both comparisons (Fig. 5B; black circle). Interestingly, for three pathways, actin cytoskeleton signaling, ERK/MAPK signaling, and G-protein coupled receptor signaling, it is predicted that these particular pathways are activated due to phosphorylation in HCM_Sarc_ versus control samples but inactivated in HCM_Neg_ compared to controls (Fig. 5D; Supplemental Table S3).

**Figure 6:**
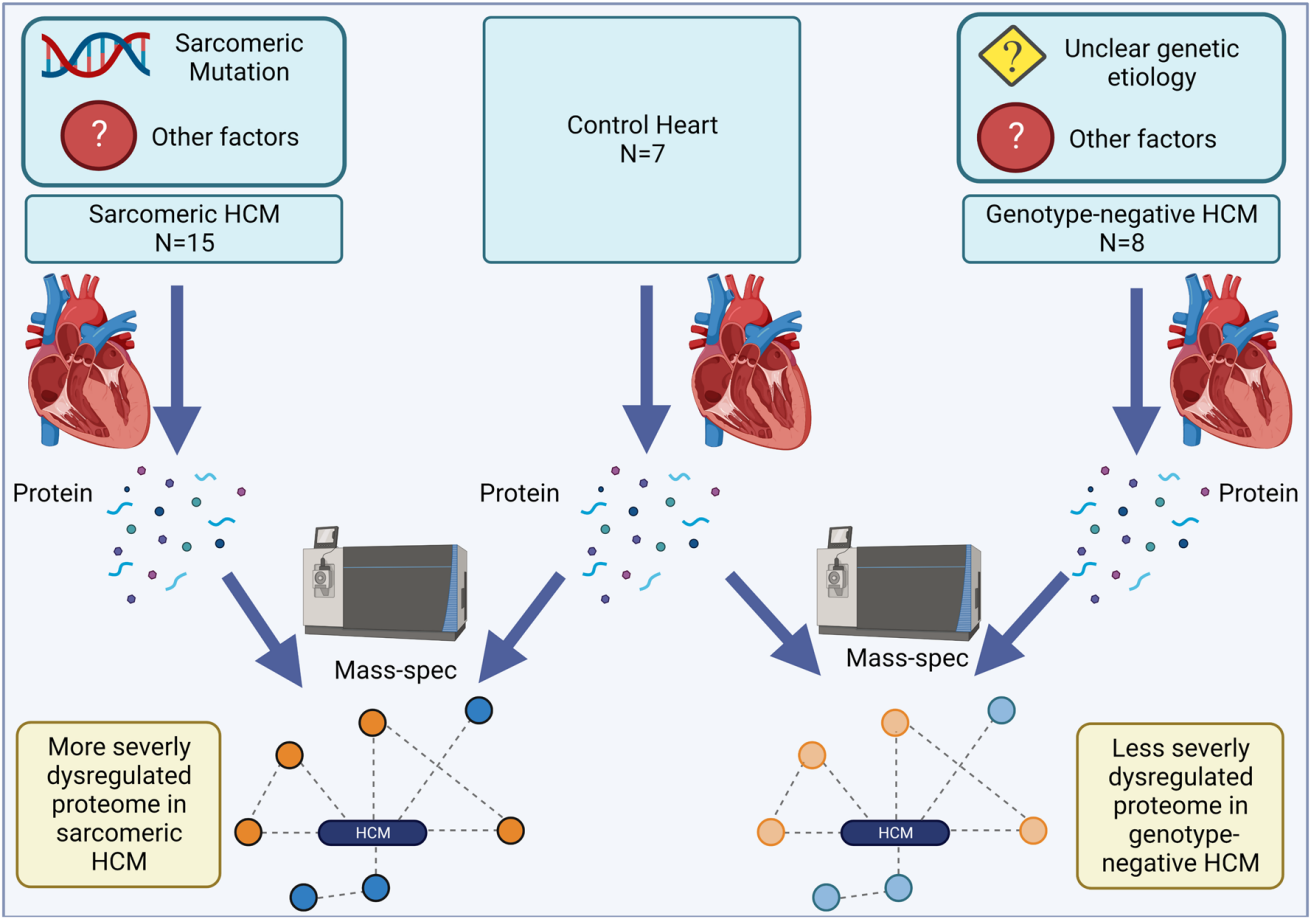
Proteome of sarcomere-positive HCM and sarcomere-negative HCM have differences in disease specific pathways with most differences between them being more severe in sarcomere-positive HCM.

Finally, when looking at the pathways that were either up- or down- regulated when directly comparing HCM_Sarc_ and HCM_Neg_ (Fig. 3B) those pathways were not only altered when comparing disease with controls (Fig. 3 and Supplemental Table S2), but the direction also matched that observed in disease. Thus HCM_Sarc_ and HCM_Neg_ have alterations in disease specific pathways and the dysregulation at least for several pathways is more severe in HCM_Sarc_ (Fig. 6).

### Comparing the two most common genotypes of sarcomeric HCM: MYBPC3-HCM and MYH7-HCM

The proteomes and phosphoproteomes for the two most common HCM-susceptibility genes: *MYBPC3* and *MYH7*, were compared. Details are provided in the Supplemental Data. The proteome of the two different HCM_Sarc_ genotypes did not separate on PCA plotting and no DEPs or DPPs were identified between the two genotypes. Thus, there is little difference in the proteomes of MYBPC3-HCM and MYH7-HCM.

## Discussion

While treatments and outcomes for HCM are improving, its vast genotypic and phenotypic heterogeneity continues to make optimal treatments and sudden cardiac death prevention challenging. Furthermore, there are enormous alterations that occur in the proteome of HCM compared with healthy controls^8–10,12^ making it hard to identify therapeutic targets. Additionally, the mono/oligo/polygenic basis or non-genetic basis for the patients classified as HCM_Neg_ remains unknown, but there is growing evidence for some within this subset to be driven by either oligogenics, epigenetics, or environmental factors, or a combination of the above^13,14^. Previous studies have shown that HCM_Sarc_ is characterized by earlier onset of disease and more severe hypertrophy^4,5^. Given these differences, there is a need for a deeper understanding of the underlying mechanisms responsible as this could help in the identification of novel therapeutic targets that modify disease progression and may help with prioritization of therapeutic targets.

Given the clinical differences across HCM genotypes^15^, there have been several studies aimed at understanding mechanistic and structural differences across genotypes which have identified nuanced differences between different genotypes^8,16–18^. While some mechanisms underlying these genotype-based differences have been elucidated, genotype comparisons of the (phospho)proteome of HCM have been limited. Using unsupervised machine learning methods, prior studies have demonstrated the proteome of HCM is similar regardless of genotype^7,8,19^ suggesting that regardless of underlying genetic etiology, HCM converges on a final common pathway. However, one of these studies, by Schuldt et al.^8^ identified that some proteins were in fact altered in HCM_Sarc_ compared with controls but not in HCM_Neg_ and vice-versa, suggesting there may in fact be subtle genotype-based differences in the proteome of HCM. However, this study did not identify proteins directly altered when comparing the two predominant HCM subtypes: those with a positive genetic test (HCM_Sarc_) and those with a negative genetic test (HCM_Neg_) and did not identify changes in hypertrophy pathways. Additionally, a second study looking at post-translational modifications and alternative splicing of sarcomeric proteins found minor genotype differences but an overall similar proteomic profile^19^. In the current study, consistent with previous proteomic studies, we found the proteomes of HCM genotypes are not distinct enough to be separated by unsupervised clustering analyses, however, our analysis did reveal direct differences in protein levels between HCM_Sarc_ and HCM_Neg_. These proteomic changes were predicted to have significant effects on cardiac hypertrophy, cardiac function, and development of heart failure. Interestingly, most of the proteins and pathways altered between HCM_Sarc_ and HCM_Neg_ were also altered when comparing HCM to controls, and in fact at the protein level, the alterations were generally more severe in HCM_Sarc_ suggesting genotype differences between HCM genotypes may be due to degree of dysregulation of the disease associated pathways.

Interestingly, at the protein level, cytoskeletal processes and extra-cellular matrix processes were significantly up-regulated when comparing HCM_Sarc_ with HCM_Neg_. This is consistent with the observation that HCM_Sarc_ shows increased fibrosis compared with HCM_Neg_ correlating with late gadolinium enhancement (LGE) which is associated with increased fibrosis on cardiac MRI^20,21^. Additionally, we observed activation of RhoA signaling and found it to be even more activated in HCM_Sarc_ compared to HCM_Neg_. RhoA signaling activates ROCK signaling which effects several cardiac cell types leading to altered cardiomyocyte contraction, cardiac gene expression, and protein phosphorylation and subsequently to cardiac hypertrophy and fibrosis^22–24^. Up-regulation of RhoA signaling and integrin signaling influences stress induced hypertrophy and is protective against heart failure; however, activation can also result in fibrosis^25^. Rho A signaling has a complex role in cardiac function and whether it is beneficial or deleterious depending on temporal and contextual factors and of cell type^25,26^. Our observations suggest that Rho A signaling has a central role in HCM as a mechanism for disease and possible cause for genotype-based differences, although further studies are necessary to parse out Rho A signaling’s precise role.

Other studies of this kind have shown HCM is characterized by widespread down-regulation of metabolic pathways, especially aerobic respiration and fatty acid oxidation^27,28^. In this study we not only replicated these findings, but also demonstrated genotype-specific differences with a more severe down-regulation of metabolic pathways observed in HCM_Sarc_. Metabolic reprogramming is observed in other forms of pathologic cardiac hypertrophy such as pressure overload hypertrophy and can serve as a marker for early onset of heart failure^29^. Consistent with our findings, down-regulation of amino acid metabolism and fatty acid metabolism and aerobic respiration have been observed as early markers towards heart failure. Thus, the more severe derangement in the proteome of HCM_Sarc_ reflects a more severe underlying metabolic reprogramming likely playing a role in genotype specific clinical differences. Additionally, we identified almost four hundred proteins altered exclusively in HCM_Sarc_ which allowed us to generate a HCM_Sarc_ protein–protein network revealing enrichment in metabolic processes. The findings suggest specific metabolic pathways may explain genotype specific differences observed in HCM and warrant further investigation.

In addition to the proteomic differences, this study showed a phosphorylation-mediated activation of integrin and cytoskeletal signaling and phosphorylation-mediated down-regulation of metabolic pathways in HCM_Sarc_. As changes in the same pathways were also observed on the protein level, this would be a strong indication that phosphorylation is regulating the function of these pathways.

Since predominant down-regulation of metabolic pathways appears central to HCM both in the proteome and phosphoproteome counteracting these changes could be a therapeutic strategy. For instance, a kinase inhibitor screen has shown promise in identifying novel therapeutics for dilated cardiomyopathy which is also characterized by metabolic reprogramming and so a similar strategy manipulating phosphorylation of metabolic pathways altered in HCM could be successful^30^. Consequently, the functional and temporal effects of protein phosphorylation warrant further investigation and could open the door to identifying novel strategies to modifying disease progression.

The phosphoproteomic differences were more complex than the proteomic changes. In some cases, phosphorylation changes were more severe in HCM_Neg_ with predominant phosphorylation-mediated inactivation. In addition, for some pathways, there were differences in the direction of change between genotypes. In contrast with HCM_Sarc,_ HCM_Neg_ showed inactivation of growth pathways such as ERK/MAPK signaling and cardiac hypertrophy signaling due to changes in phosphorylation. As we identified previously, this signaling cascade appears central to many hypertrophy pathways up-regulated in HCM^7^. This phosphorylation-mediated inactivation could contribute to the less severe progression observed in HCM_Neg_.

### Limitations

This study was performed on myectomy tissues and thus provides a snapshot view of the myocardium at the time of myectomy, representing obstructive HCM generally in a later stage of disease progression. Therefore, these identified perturbations in the (phospho)proteomic architecture of obstructive HCM may not apply to the other morphologic subtypes of HCM such as non-obstructive HCM or apical HCM.

## Conclusion

Although the proteome and phosphoproteome of obstructive HCM is similar regardless of genetic etiology, there were important differences between HCM_Sarc_ and HCM_Neg_ with a more severe dysregulation of disease relevant pathways observed in HCM_Sarc_. Further studies are necessary to determine whether these changes underlie the clinical differences between HCM_Sarc_ and HCM_Neg_.

### Supplementary Information

Supplementary Information 1.

Supplementary Figure 1.

Supplementary Figure 2.

Supplementary Figure 3.

Supplementary Figure 4.

Supplementary Figure 5.

Supplementary Figure 6.

Supplementary Figure 7.

Supplementary Information 2.

## Data Availability

Proteomic and phosphoproteomic data are available at the MassIVE database (MSV000091821 and MSV000091822, respectively).

## References

[1] Ingles J (2019). Evaluating the clinical validity of hypertrophic cardiomyopathy genes. Circ. Genom. Precis. Med..

[2] Bos JM (2014). Characterization of a phenotype-based genetic test prediction score for unrelated patients with hypertrophic cardiomyopathy. Mayo Clin. Proc..

[3] Marian AJ, Braunwald E (2017). Hypertrophic cardiomyopathy: Genetics, pathogenesis, clinical manifestations, diagnosis, and therapy. Circ. Res..

[4] Olivotto I (2008). Myofilament protein gene mutation screening and outcome of patients with hypertrophic cardiomyopathy. Mayo Clin. Proc..

[5] Ho CY (2018). Genotype and lifetime burden of disease in hypertrophic cardiomyopathy: Insights from the Sarcomeric Human Cardiomyopathy Registry (SHaRe). Circulation.

[6] Bos JM, Ommen SR, Ackerman MJ (2007). Genetics of hypertrophic cardiomyopathy: One, two, or more diseases?. Curr. Opin. Cardiol..

[7] Garmany R (2023). Multi-omic architecture of obstructive hypertrophic cardiomyopathy. Circ. Genom. Precis. Med..

[8] Schuldt M (2021). Proteomic and functional studies reveal detyrosinated tubulin as treatment target in sarcomere mutation-induced hypertrophic cardiomyopathy. Circ. Heart Fail..

[9] Shimada YJ (2019). Application of proteomics profiling for biomarker discovery in hypertrophic cardiomyopathy. J. Cardiovasc. Transl. Res..

[10] Coats CJ (2018). Proteomic analysis of the myocardium in hypertrophic obstructive cardiomyopathy. Circ. Genom. Precis. Med..

[11] Richards S (2015). Standards and guidelines for the interpretation of sequence variants: A joint Consensus Recommendation of the American College of Medical Genetics and Genomics and the Association for Molecular Pathology. Genet. Med..

[12] Shimada YJ (2021). Comprehensive proteomics profiling reveals circulating biomarkers of hypertrophic cardiomyopathy. Circ. Heart Fail..

[13] Ho CY (2015). Genetic advances in sarcomeric cardiomyopathies: State of the art. Cardiovasc. Res..

[14] Teekakirikul P, Zhu W, Huang HC, Fung E (2019). Hypertrophic cardiomyopathy: An overview of genetics and management. Biomolecules.

[15] Geske JB, Ommen SR, Gersh BJ (2018). Hypertrophic cardiomyopathy: Clinical update. JACC Heart Fail..

[16] Witjas-Paalberends ER (2014). Gene-specific increase in the energetic cost of contraction in hypertrophic cardiomyopathy caused by thick filament mutations. Cardiovasc. Res..

[17] Vakrou S (2021). Differences in molecular phenotype in mouse and human hypertrophic cardiomyopathy. Sci. Rep..

[18] Captur G (2014). Prediction of sarcomere mutations in subclinical hypertrophic cardiomyopathy. Circ. Cardiovasc. Imaging.

[19] Tucholski T (2020). Distinct hypertrophic cardiomyopathy genotypes result in convergent sarcomeric proteoform profiles revealed by top-down proteomics. Proc. Natl. Acad. Sci. U S A.

[20] Ellims AH (2014). A comprehensive evaluation of myocardial fibrosis in hypertrophic cardiomyopathy with cardiac magnetic resonance imaging: Linking genotype with fibrotic phenotype. Eur. Heart J. Cardiovasc. Imaging.

[21] Teramoto R (2018). Late gadolinium enhancement for prediction of mutation-positive hypertrophic cardiomyopathy on the basis of panel-wide sequencing. Circ. J..

[22] Yura Y (2016). Focused proteomics revealed a novel rho-kinase signaling pathway in the heart. Cell Struct. Funct..

[23] Loirand G, Guérin P, Pacaud P (2006). Rho kinases in cardiovascular physiology and pathophysiology. Circ. Res..

[24] Shimizu T, Liao JK (2016). Rho kinases and cardiac remodeling. Circ. J..

[25] Lauriol J (2014). RhoA signaling in cardiomyocytes protects against stress-induced heart failure but facilitates cardiac fibrosis. Sci. Signal..

[26] Shimokawa H, Sunamura S, Satoh K (2016). RhoA/Rho-kinase in the cardiovascular system. Circ. Res..

[27] Previs MJ (2022). Defects in the proteome and metabolome in human hypertrophic cardiomyopathy. Circ. Heart Fail..

[28] van der Velden J (2018). Metabolic changes in hypertrophic cardiomyopathies: Scientific update from the Working Group of Myocardial Function of the European Society of Cardiology. Cardiovasc. Res..

[29] Lai L (2014). Energy metabolic reprogramming in the hypertrophied and early stage failing heart: A multisystems approach. Circ. Heart Fail..

[30] Perea-Gil I (2022). Serine biosynthesis as a novel therapeutic target for dilated cardiomyopathy. Eur. Heart J..

[31] Yu SH, Kyriakidou P, Cox J (2020). Isobaric matching between runs and novel PSM-level normalization in MaxQuant strongly improve reporter ion-based quantification. J. Proteome Res..

[32] Huang T (2020). MSstatsTMT: Statistical detection of differentially abundant proteins in experiments with isobaric labeling and multiple mixtures. Mol. Cell Proteomics.

[33] Szklarczyk D (2019). STRING v11: Protein-protein association networks with increased coverage, supporting functional discovery in genome-wide experimental datasets. Nucleic Acids Res..

