# Supplementary figures and images for: Proteomic and phosphoproteomic analyses of myectomy tissue reveals difference between sarcomeric and genotype-negative hypertrophic cardiomyopathy
Source: Sci Rep. 2023 Sep 1;13:14341. doi: 10.1038/s41598-023-40795-1 (PMC10474105; doi:10.1038/s41598-023-40795-1)

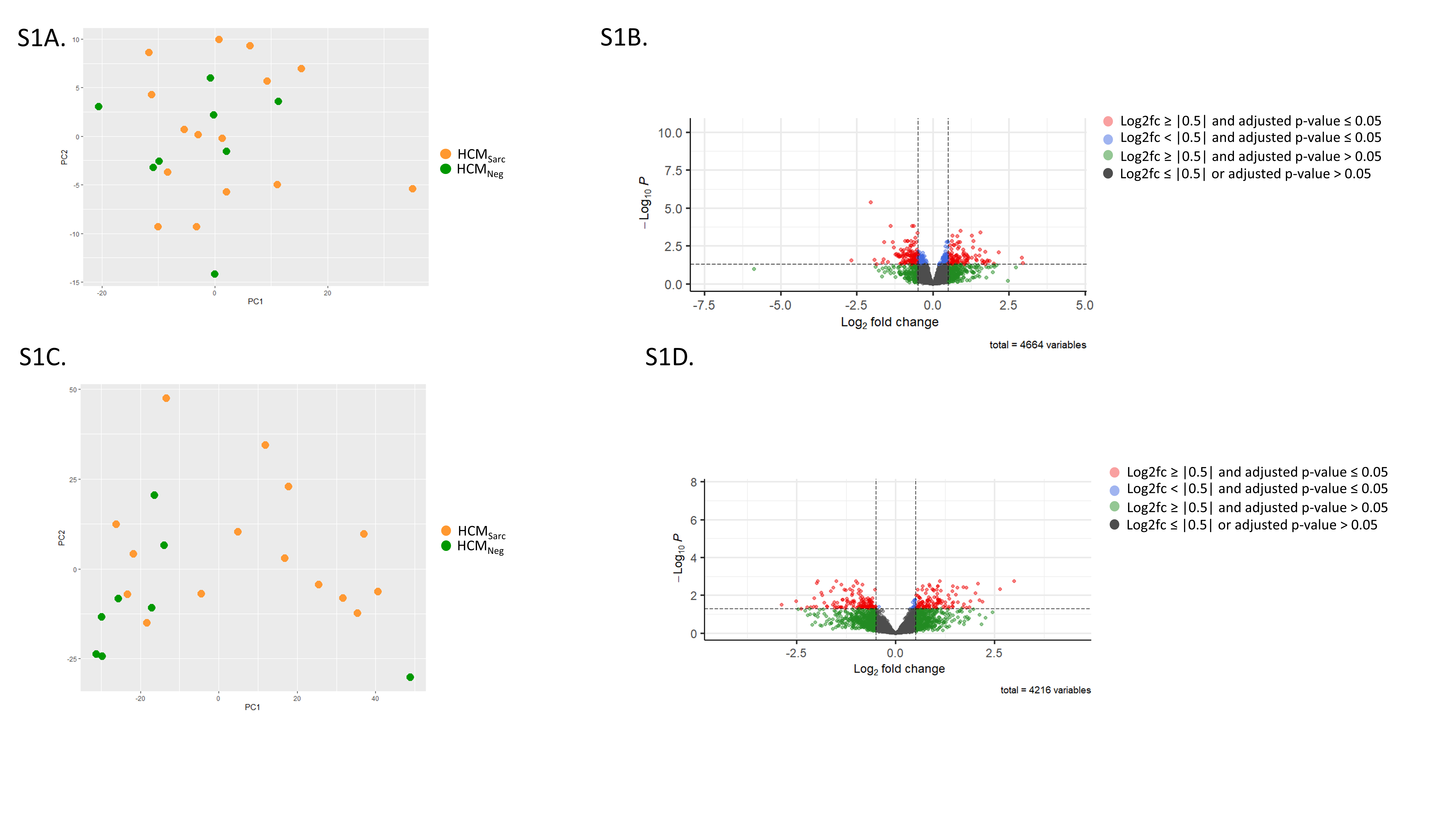

Supplement: Supplementary file 2 — Supplementary Figure 1. [file 41598_2023_40795_MOESM2_ESM.tif]

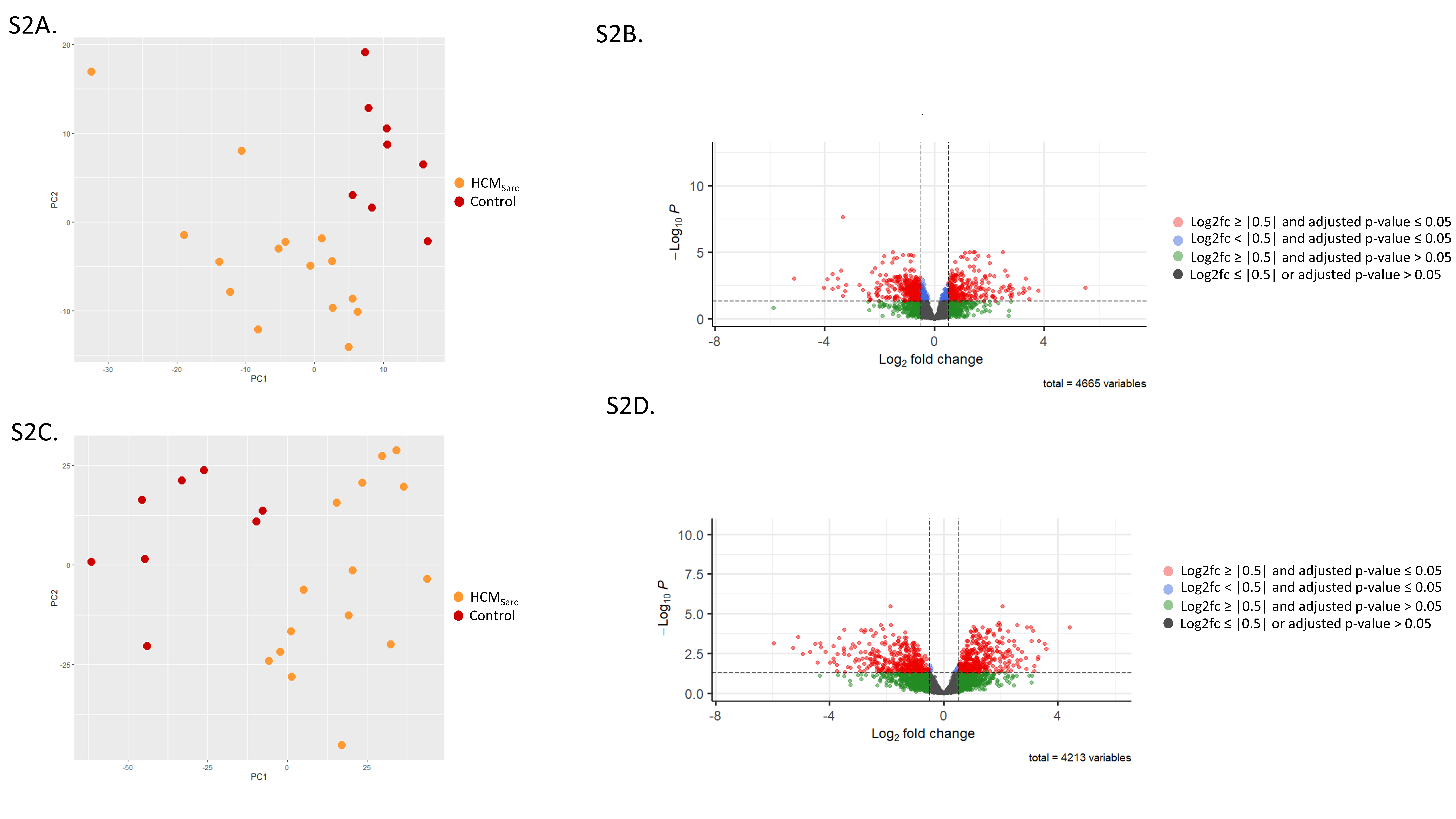

Supplement: Supplementary file 3 — Supplementary Figure 2. [file 41598_2023_40795_MOESM3_ESM.tif]

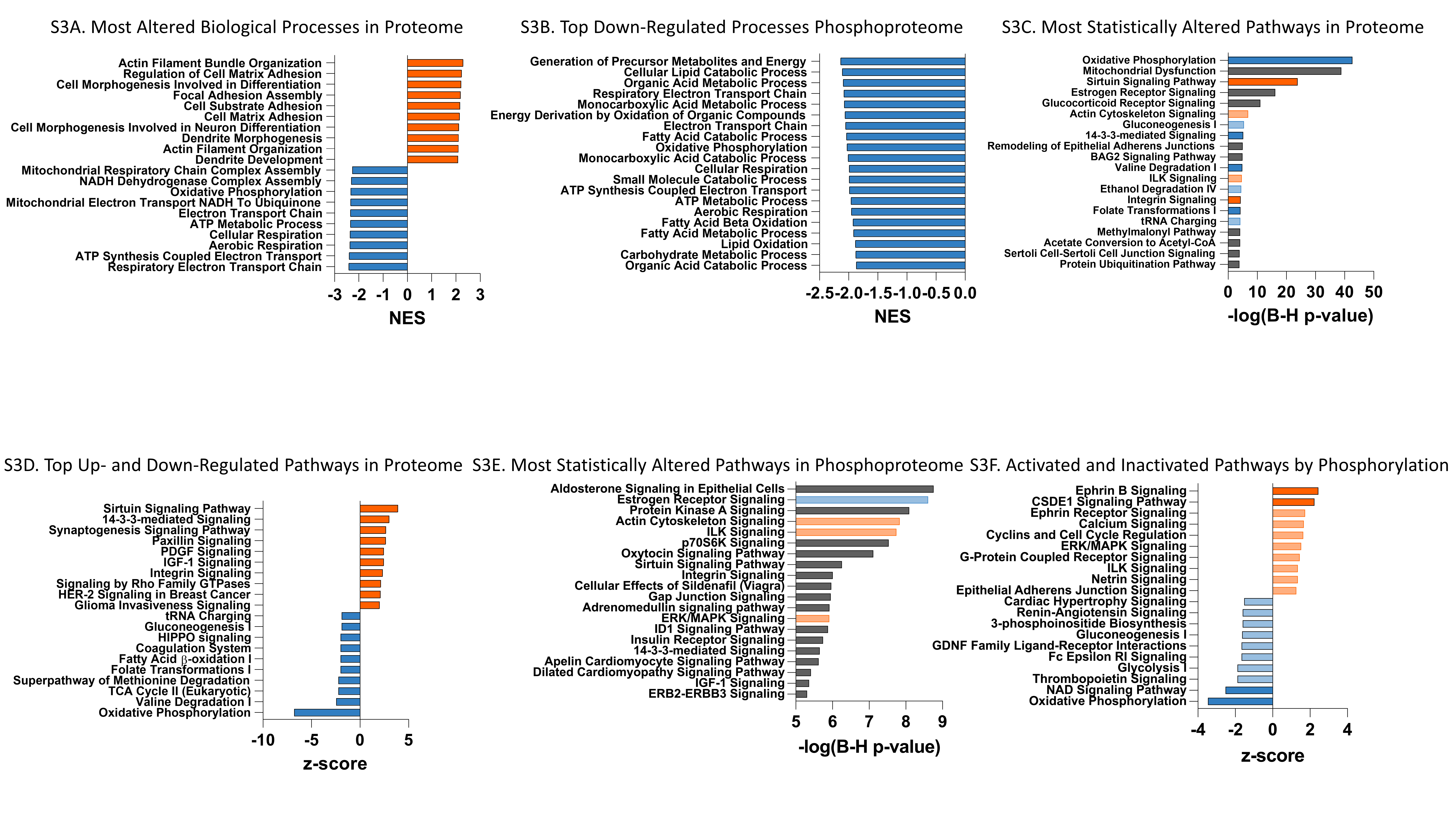

Supplement: Supplementary file 4 — Supplementary Figure 3. [file 41598_2023_40795_MOESM4_ESM.tif]

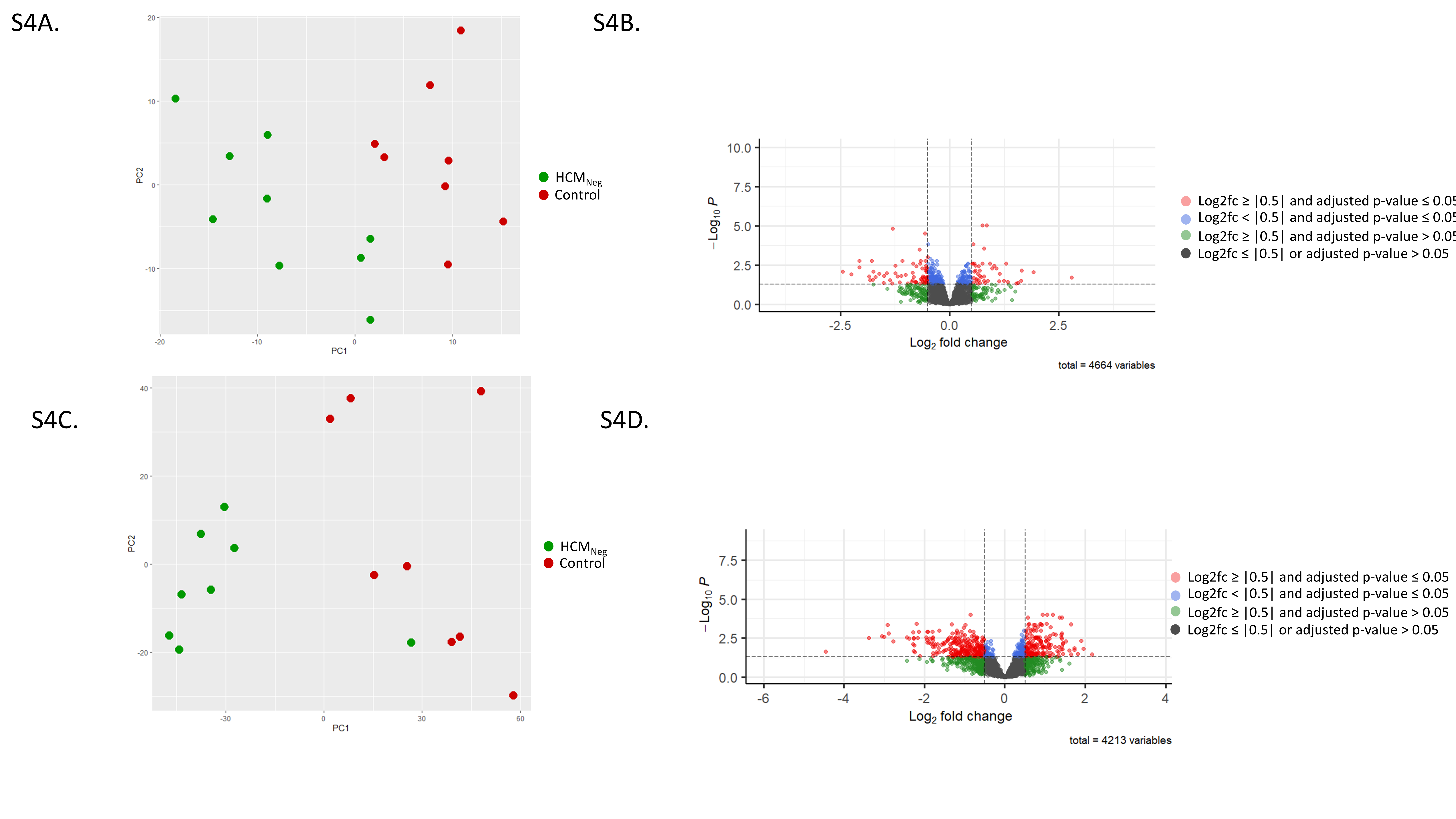

Supplement: Supplementary file 5 — Supplementary Figure 4. [file 41598_2023_40795_MOESM5_ESM.tif]

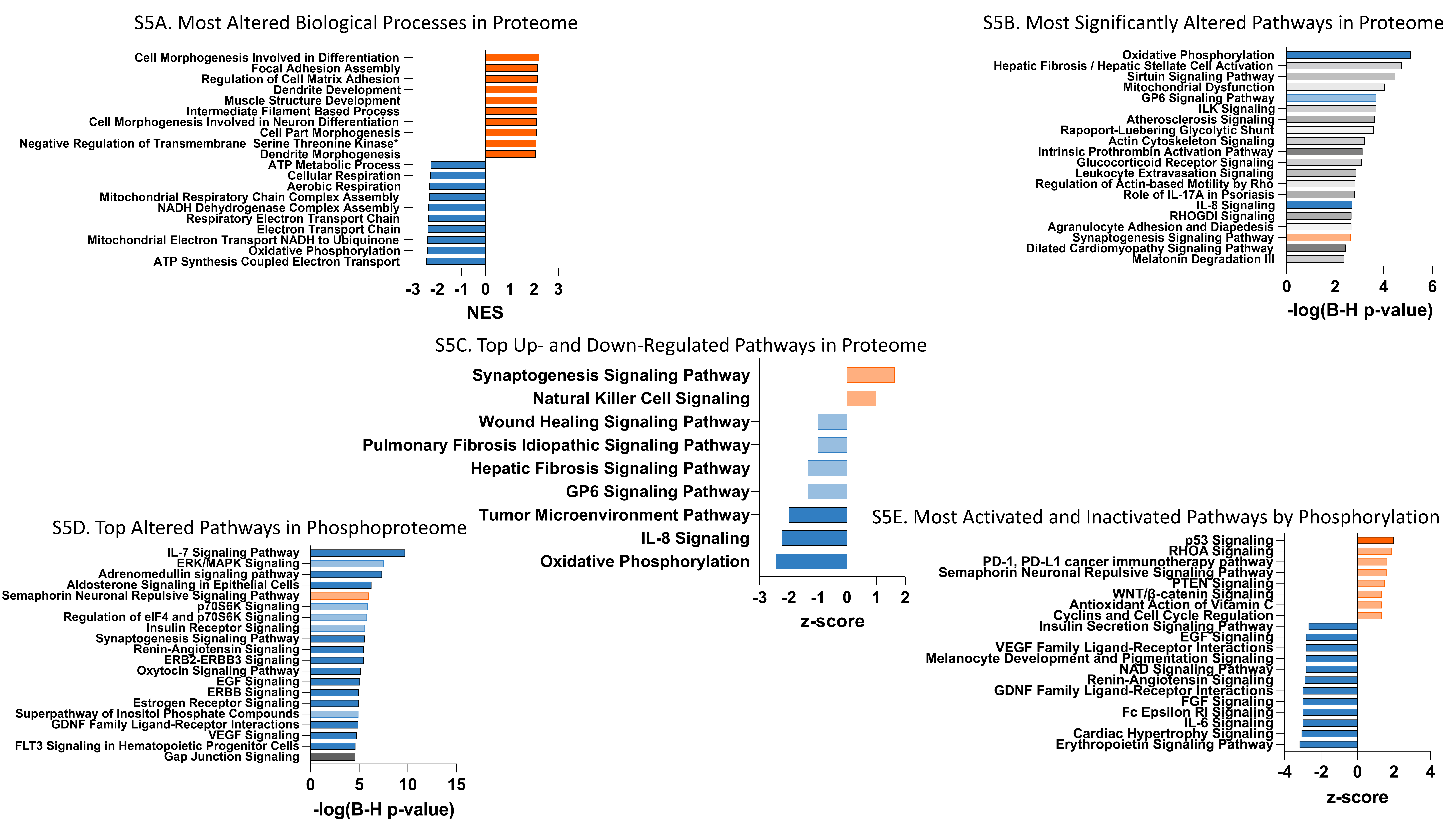

Supplement: Supplementary file 6 — Supplementary Figure 5. [file 41598_2023_40795_MOESM6_ESM.tif]

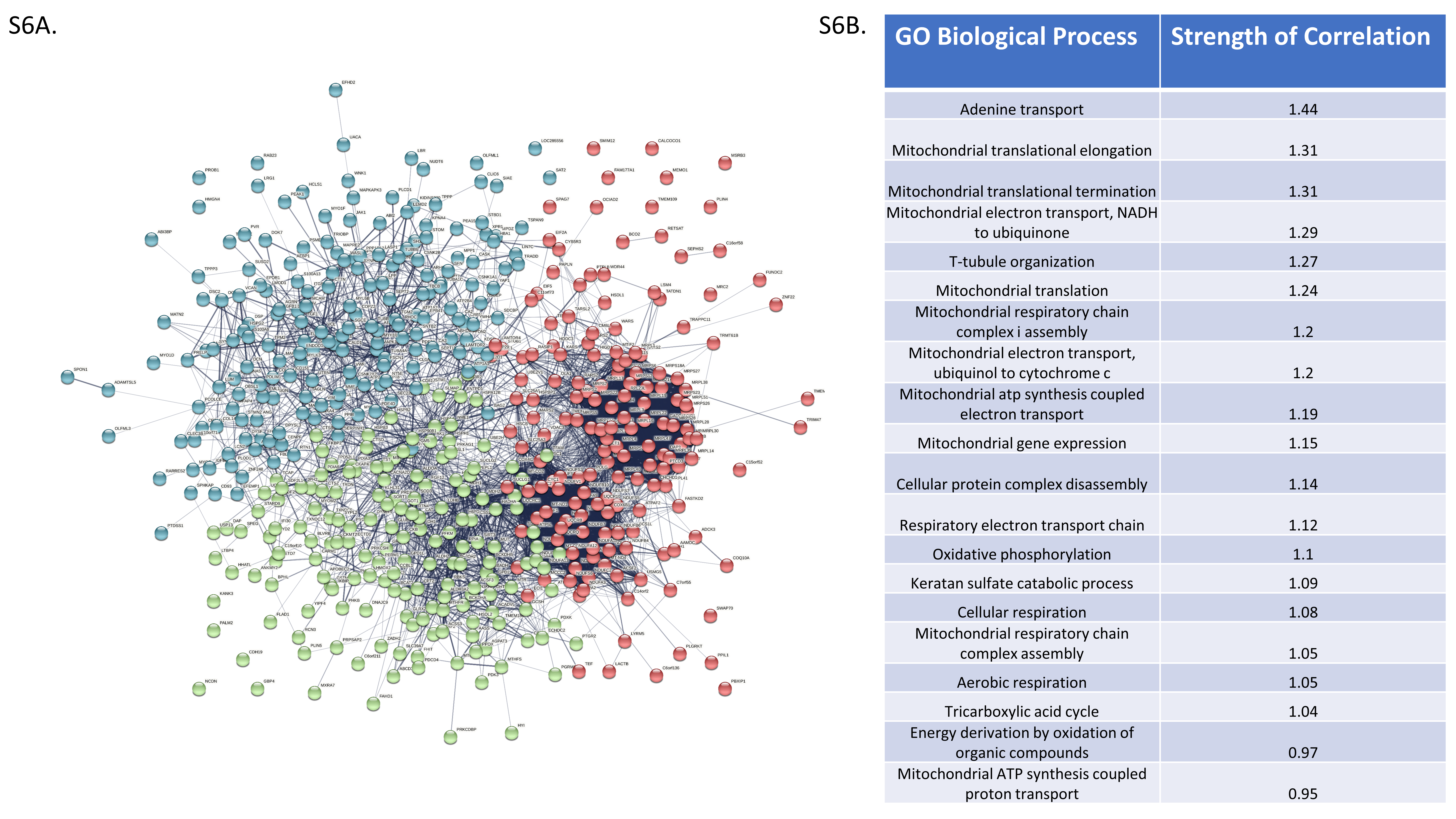

Supplement: Supplementary file 7 — Supplementary Figure 6. [file 41598_2023_40795_MOESM7_ESM.tif]

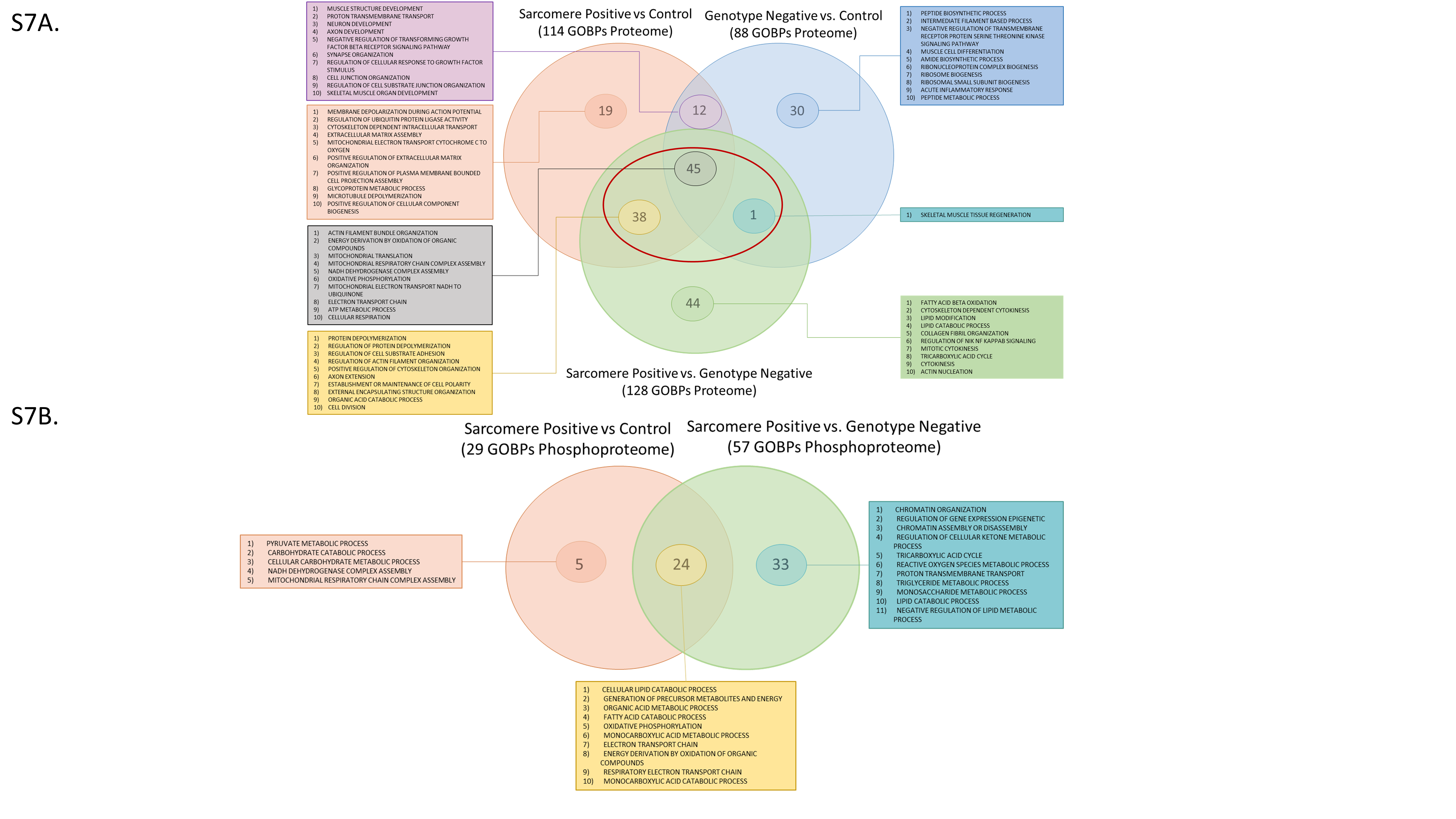

Supplement: Supplementary file 8 — Supplementary Figure 7. [file 41598_2023_40795_MOESM8_ESM.tif]
