# Supplementary material for: Proteomic and phosphoproteomic analyses of myectomy tissue reveals difference between sarcomeric and genotype-negative hypertrophic cardiomyopathy
Source: Sci Rep. 2023 Sep 1;13:14341. doi: 10.1038/s41598-023-40795-1 (PMC10474105; doi:10.1038/s41598-023-40795-1)
Supplement: Supplementary file 9 — Supplementary Information 2. [file 41598_2023_40795_MOESM9_ESM.docx]

**Supplemental Methods:**

*HCM Clinical Cohort*

This subgroup study is derived from a Mayo Clinic IRB-approved (811-98) retrospective cohort study consisting of 23 patients diagnosed with obstructive HCM needing a surgical myectomy. Myectomy tissues were flash frozen in liquid nitrogen immediately after resection in the operating room and stored at -80°C. As previously described^7^, genome sequencing was performed on all HCM patients to identify genetic variants among the 54 published HCM-associated genes. All genetic variants were classified according to the 2015 American College of Medical Genetics and Genomics criteria^11^. Patients were then divided into genotype subgroups. Sarcomere-positive HCM (HCM_Sarc_) was defined as patients with pathogenic/likely pathogenic variants in definitive, strong, or moderate evidence HCM-causative genes that encode sarcomeric proteins while genotype-negative HCM (HCM_Neg_) was defined as those without any disease-causative variants plus any variants of uncertain significance (VUS) in any of the 54 genes.

*Experimental Design and Statistical Rationale*

As previously described, mass-spectometry based proteomics and phosphoproteomics were performed on myectomy tissue of the HCM_Sarc_ (N = 15) and HCM_Neg_ (N = 8) patients along with control cardiac tissue (N = 7) from healthy cardiac donors for whom a suitable recipient was not identified^7^. All samples were used as biological replicates for each analysis. For differential expression, the peptide and protein identification data along with the respective corrected TMT intensity data were processed using MSstatsTMT^31^ package using R statistical programming environment (version 4.0). MSstatsTMT modeled the sample-wise intensities of each protein and compared them between any two groups of samples using the moderated t statistic method. Resulting protein differential expression p-values were corrected using the Benjamini-Hochberg method. Proteins with an adjusted p-value of <= 0.05 and an absolute log2 fold change of >=0.5 (where 0.0 signifies no change) were considered as statistically significant for interpretation. Details of the statistical methods used for gene set and pathway analysis are included in the respective section below.

*Sample Preparation*

Frozen tissues were pulverized with a stainless-steel mortar and pestle in dry ice (mortar/pestle put in -80°C freezer overnight prior to use, then covered in dry ice to preserve temperature during pulverization); liquid nitrogen was occasionally added to the mortar to preserve cold temperature. Powdered samples were lysed in 5% SDS RIPA buffer with protease (Roche Mini Complete Plus EDTA) and phosphatase (Pierce HALT) inhibitors by alternating sonication with brief cooling in an ice bath being careful to avoid SDS precipitation. A small aliquot of each sample was assayed for total protein content by BCA assay. One mg aliquots of samples in 500 µL lysis buffer were reduced with DTT (20 mM final concentration) at 85 °C for 10 minutes and after cooling were alkylated with 100 mM iodoacetamide in the dark at room temperature for 30 minutes. Digestion and SDS removal were done using S-trap midi-scale columns and protocol (Protifi.com, Fairport, NY). 500 µL of sample in 5% SDS RIPA buffer were acidified with 25uL of 27.5% phosphoric acid and added to 3.3 mL of S-trap buffer (90% methanol, 100mM TEAB, pH 7.5) in a 5 mL microcentrifuge tube to create a colloidal suspension of proteins. Proteins were adsorbed to S-trap columns in two loadings, each followed by centrifugation for 1 minute at 3500 x g. Samples were then washed four times with 3 mL of S-trap buffer to remove SDS. Digestions were performed on the S-trap columns by adding 50 µg of Lys-C/trypsin (Promega) in 350 µL of 50mm TEAB, pH 8.5 to the top of the column bed for each sample. Samples were centrifuged 1min at 200 rpm to distribute trypsin solution into the columns, followed by incubation for one hour at 47°C in a thermomixer with heated lid (Eppendorf). After digestion, peptides were serially eluted with 500 µL of 50 mM TEAB, 500 uL of 0.2% formic acid, and 500 µL of 50% acetonitrile in 0.2% formic acid, each addition followed by spinning 1min. at 4000 x g. The three elutions were combined in a 15mL centrifuge tube, frozen at -80°C, and lyophilized to dryness.

*Tandem Mass Tag Labeling*

Lyophilized samples were multiplexed for mass spec analysis by reacting peptides with isobaric Tandem Mass Tag reagents (TMTpro 16-plex, Thermo Fisher Scientific). Labeling was performed in two batches with one control sample being used in both TMT batches as a bridge sample. The bridge sample was prepared from dual 1 mg aliquots of the lysate that had been carried separately through the S-trap cleanup and digestion process. Prior to TMT tagging, the two bridge samples were mixed and re-split to create the two bridge samples for TMT labeling. Samples were reacted with their TMT reagent containing unique reporter ions.

*Fractionation of TMT-labeled Samples and Enrichment of Phosphorylated peptides*

The two multiplexed TMT sets, each containing 12 mg of multiplexed peptides, were each fractionated by basic pH reversed phase liquid chromatography (bRPLC) on a 250 mm x 4.6mm i.d., 3.5um, Waters BEH C_18_ peptide column (Waters, Milford, MA) with mobile phase A being 5 mM ammonium formate, pH 8.5 and mobile phase B being 80% acetonitrile 5mM ammonium formate, pH 8.5. Peptides were separated using an 80 min. LC method with a gradient from 5% B to 40% B over 60 min at 0.5 mL/min. 96 fractions were collected over the retention time from 4 minutes to 80 minutes (47s fractions), and the 96 fractions were re-combined in a staggered pattern to 12 fractions. The combined fractions were split three ways with 128 ug being set aside for total proteome analysis, 2.4 mg allocated for metal ion enrichment of phosphopeptides.

Phosphopeptide enrichment was performed on an Agilent AssayMap Bravo automated liquid handler using Fe-NTA metal affinity cartridges. After initial cleaning and equilibration of the Fe-NTA cartridges, peptides were adsorbed to the cartridge in 80% acetonitrile/0.4% TFA and washed with 0.1% TFA in acetonitrile to remove non-phosphorylated peptides. Phosphopeptides were recovered by serial 25 µL elutions of 5% ammonium hydroxide, followed by 50% acetonitrile with 5% ammonium hydroxide into a 96-well plate containing 15µL of 10% formic acid. Eluates were transferred to autosampler vials, dried with a vacuum centrifuge, and stored at -80°C until mass spectrometry analysis.

LC-MS/MS Methods

Nano-scale liquid chromatography tandem mass spectrometry(nLC-MS/MS) analyses of the sample fractions were performed with a Dionex 3000 Ultimate nano-scale liquid chromatograph in tandem with an Orbitrap Exploris480 mass spectrometry (Thermo Fisher Scientific, Danvers, MA). Samples were reconstituted in an aqueous solution of 0.2% formic acid, 0.1% TFA, and 0.0005% Zwittergent 3-16 and preconcentrated via autosampler on a 0.33 µL Halo Peptide ES-C_18_ Exp2 stem trap (Optimize Technologies, Oregon City, Oregon) and separated on a 35 cm x 100 μm i.d. column packed in-house with Acclaim RSLC 2.2 μm C_18_ (Thermo Fisher) heated to 40 ºC. A 120 min gradient with 0.4 μL/min flow rate was used from 2% to 40% mobile phase B where mobile phase A was 2% acetonitrile in 0.2% formic acid and mobile phase B was 80% acetonitrile, 10% isopropanol, 10% water and 0.2% formic acid overall. The mass spectrometer was operated in a data-dependent acquisition (DDA) mode where MS1 survey scans were measured with 120,000 resolving power at m/z 200) with automatic gain control (AGC) = 3e6, max. ion fill time = 100 ms. Tandem mass spectra (MS2) were acquired on precursor ions with charge of 2-4, using MS2 resolving power = 45,000 (at m/z 200), normalized collision energy (NCE) = 32%, quadrupole isolation width = 0.7, AGC = 2E5, and max. fill time = 105 ms.

*Global proteome identification and differential expression*

We utilized an established workflow^32^ for processing the TMT 16-plex raw data to identify proteins and perform differential expression. For this, raw data files from the two TMT 16-plex experiments were processed using MaxQuant (version 2.0.3.0) software configured to use SwissProt human protein sequence database (Uniprot.org, downloaded on 03/2021) augmented with common contaminant proteins (cotton, wool, etc.). Reversed protein sequences were appended to the database to estimate the protein and peptide identification false discovery rates (FDRs). The software was configured to derive fully tryptic peptides from the database and utilize default orbitrap mass tolerances when performing peptide-spectrum matching. MaxQuant was also configured to use the following variable modifications: carbamidomethylation of cysteine, oxidation of methionine, deamidation of asparagine and TMT 16-plex labeling reagent specific modifications. A maximum of four modifications per peptide were allowed. Software was instructed to combine results from all fractions of both TMT 16-plex experiments and identify proteins with an FDR of 1%. MaxQuant extracted the sample-wise TMT intensities of the identified proteins using a precursor ion purity of >=0.9 and corrected them to account for the TMT channel impurity.

The peptide-spectrum matches were loaded into the package and sample-wise protein intensity data was generated by using both global intensity normalization (to account for intensity variation within each TMT 16-plex experiment) and reference channel normalization (to account for intensity variation between the two TMT 16-plex experiments). Normalized sample-wise intensity data of all proteins was utilized to find differentially expressed proteins between any two groups of interest.

*Phosphoproteomics identification and quantification*

The phosphoproteomics TMT 16-plex raw data were processed using the above-described method with following modifications. MaxQuant was configured to allow for the following additional variable modifications: phosphorylation of serine, threonine, and tyrosine. The number of variable modifications allowed per peptide was also increased to five. The resulting phosphopeptide identification and corrected TMT intensity data were processed using MSstatsTMT following a slightly modified process that was described above. First, the sample-wise phosphopeptide intensity data was summarized into sample-wise phosphoprotein intensities. Resulting sample-wise phosphoprotein intensities were compared across any two groups following the above-described method. In parallel, the phosphopeptides and their corresponding sample-wise intensities were compared between any two groups using the same moderated t statistic as described above. Phosphoproteins and phosphopeptides with an adjusted differential expression p-value of <=0.05 and an absolute log2 fold change >=0.5 (where 0.0 signifies no change) were considered as statistically significant for further interpretation.

*Pre-Ranked Gene Set Enrichment Analysis (GSEA)*

The genes representing proteins were ranked using the formula -1*log (differential expression p-value)*sign(fold change) and inputted into the Broad’s Gene Set Enrichment software (version 4.0.0) to perform enrichment analysis of biological processes. A minimum gene set size of 5 and a maximum gene set size of 1500 were used during the analysis. Enrichment sets with an FDR ≤ 0.05 were considered as significant.

*Ingenuity Pathway Analysis*

DEPs and DPPs were inputted into Ingenuity Pathway Analysis (IPA; Qiagen). Pathways were considered statistically altered if Benjamini-Hochberg (BH) corrected p-value of ≤ 0.05 (-log [BH p-value] ≥ 1.3). Pathways with a z-score ≥ 1 were considered up-regulated for DEPs or activated for DPPs and those with a z-score ≤ 1 were considered down-regulated for DEPs or inactivated for DPPs.

*Generation of Sarcomere-Positive Protein Network*

All the proteins identified to be altered in sarcomere-positive HCM, but not genotype-negative HCM were inputted into STRINGS (Version 11.5) functional protein-protein association network^33^ and the network and enrichment data were exported.

**Supplemental Results**

***Comparison of* HCM_Sarc_ *versus Controls***

To observe whether the changes between HCM_Sarc_ and HCM_Neg_ occur in pathways that are altered in disease state, we subsequently performed comparisons of each genotype-subgroup with controls. As anticipated HCM_Sarc_ demonstrated clear differences compared to control samples (**Supplemental Figure 2A**) with a total of 664 DEPs: 267 up-regulated and 397 down-regulated (**Supplemental Figure 2B**). Additionally, the phosphoproteome was also distinct from controls (**Supplemental Figure 2C**) with 772 DPPs: 378 hypophosphorylated and 394 hyperphosphorylated (**Supplemental Figure 2D**). There were 114 GO biological processes altered in the proteome of HCM_Sarc_ compared with controls with a predominant up-regulation of cytoskeletal and extracellular matrix processes and down-regulation of metabolic processes (**Supplemental** **Figure 3A**). At the phosphoproteome, 29 processes were down-regulated with all of them being metabolic (**Supplemental** **Figure 3B)**.

A total of 124 pathways were altered at the proteome level with 25 up-regulated (z-score ≥ 1), 21 down-regulated (z-score ≤ -1), and 78 for which directionality could not be determined. Pathway analysis of HCM_Sarc_ compared with controls showed similarities with HCM_Sarc_ compared with HCM_Neg_. **Supplemental Figure 3C** summarizes the most statistically altered pathways while **Supplemental** **Figure 3D** summarizes the pathways with the largest predicted effect sizes (z-scores). Interestingly the two pathways most up-regulated in HCM_Sarc_ versus controls were also altered when comparing HCM_Sarc_ versus HCM_Neg_ (Supplement). In addition, two pathways that were prominently up-regulated in HCM_Sarc_ compared with HCM_Neg_ are up-regulated compared to controls as well with signaling by Rho Family GTPases (z-score = 2.1) being among the most up-regulated pathways (**Supplemental** **Figure 3C**) and Rho A signaling being moderately up-regulated (z-score = 1.7; Supplement). In addition, many metabolic pathways down-regulated in the genotype comparison were also down-regulated in HCM_Sarc_ compared with controls (**Supplemental Figure 3D**). Pathway analysis of the changes in protein phosphorylation revealed 220 pathways altered due to changes in phosphorylation with 20 activated, 38 inactivated, and 162 with an unclear effect. The pathways most affected (-log[BH p-value]) by changes in phosphorylation are shown in **Supplemental Figure 3E** and those with the greatest predicted effect size (z-score ≥ |1|) are shown in **Supplemental Figure 3F**. Many of the up-regulated pathways in the proteome were also predicted to be activated due to phosphorylation such as calcium signaling (z-score = 1.7), ERK/MAPK signaling (z-score = 1.5), G-protein coupled receptor signaling (z-score = 1.4), and ILK signaling (z-score = 1.3). Additionally, many down-regulated pathways in the proteome were predicted to be inactivated due to phosphorylation as well such as oxidative phosphorylation (z-score = -3.5), glycolysis I (z-score = -1.9), gluconeogenesis (z-score = -1.6), and cardiac hypertrophy signaling (z-score = -1.6). Thus, widespread changes in disease related pathways occur due to both changes in protein levels and altered levels of phosphorylation.

***Comparison of the* HCM_Neg_ *and Controls***

The proteome of HCM_Neg_ cohort was also distinct from controls with 109 DEPs; 42 up-regulated and 67 down-regulated (**Supplemental Figure 4A**) and clear separation on PCA plotting (**Supplemental** **Figure 4B**). In addition, the phosphoproteome of HCM_Neg_ is distinct from controls (**Supplemental Figure 4C**) with 458 DPPs: 271 hypophosphorylated and 187 hyperphosphorylated (**Supplemental Figure 4D**). A complete list of DPPs is included in the **data supplement**.

GSEA identified 88 GO biological processes that were altered in HCM_Neg_ compared with control with 58 up-regulated and 30 down-regulated at the proteome level (**Supplemental** **Figure 5A**). The main biological processes up-regulated were involved in cytoskeletal and extracellular matrix and the main down-regulated biological processes were metabolic especially aerobic respiration and mitochondrial processes. There were no significantly altered processes in the phosphoproteome.

Pathway analysis of the proteome identified 74 pathways altered between HCM_Neg_ and controls with 2 up-regulated (z-score ≥ 1), 7 down-regulated (z-score ≤ -1), and 65 with indeterminate directionality. The most statistically altered pathways are summarized in **Supplemental** **Figure 5B** while the pathways with the greatest predicted effect size (z-score) are summarized in **Supplemental** **Figure 5C**. Pathway analysis revealed alterations in 180 pathways due to phosphorylation with 8 being activated, 115 inactivated, and 57 with unclear directionality. The most altered pathways (-log[B-H p-value]) are presented in **Supplemental** **Figure 5D**. Of note, the most altered pathways were almost entirely predicted to be inactivated due to phosphorylation (z-score ≤ 1). The top activated (z-score ≥1) and inactivated (z-score ≤ 1) pathways are presented in **Supplemental** **Figure 5E**

**Supplemental Table 1. Demographic and Clinical Information for Entire HCM Cohort.**

|  | **HCM_Sarc_**  (n = 43) | **HCM_Neg_**  (n = 11) | p-value |
| --- | --- | --- | --- |
| Female, n(%) | 22 (51%) | 6 (55%) | 0.8 |
| Average age at myectomy, y | 40±17 | 52±16 | 0.02 |
| Average age at diagnosis, y | 33±16 | 47±19 | 0.04 |
| Family Hx of HCM, n(%) | 24 (56%) | 2 (18%) | 0.04 |
| Family Hx of SCA, n(%) | 11 (26%) | 2 (18%) | 0.9 |
| SCA, n(%) | 1 (2%) | 0 | 0.9 |
| ICD, n(%) | 12 (28%) | 1 (10%) | 0.3 |
| Max LVWT at diastole, g/m^2^ | 24±8 | 21±7 | 0.3 |
| Max LVOT gradient, mmHg | 80±39 | 91±52 | 0.5 |

**HCM, hypertrophic cardiomyopathy; ICD, implantable cardioverter-defibrillator; LV, left ventricle; LVOT, left ventricular outflow tract; LVWT, left ventricular wall thickness; Max, maximum; SCA, sudden cardiac arrest.**

**Supplemental Table 2.** Directionality of Pathways (based on z-score) Altered in the Proteomes of Both Sarcomere-Positive HCM and Genotype-Negative HCM Compared with Controls.

| Ingenuity Canonical Pathways | Sarcomere-Positive HCM versus Control | Genotype-Negative HCM versus Control |
| --- | --- | --- |
| Acetate Conversion to Acetyl-CoA | ND | ND |
| Actin Cytoskeleton Signaling | 1.213 | 0.447 |
| Agrin Interactions at Neuromuscular Junction | 1.633 | ND |
| Apelin Liver Signaling Pathway | ND | ND |
| Arsenate Detoxification I (Glutaredoxin) | ND | ND |
| BAG2 Signaling Pathway | 0.302 | ND |
| Calcium Signaling | 1.633 | ND |
| Cellular Effects of Sildenafil (Viagra) | ND | ND |
| Clathrin-mediated Endocytosis Signaling | ND | ND |
| Creatine-phosphate Biosynthesis | ND | ND |
| Dilated Cardiomyopathy Signaling Pathway | 0 | ND |
| Estrogen Receptor Signaling | 0.775 | ND |
| Fcγ Receptor-mediated Phagocytosis in Macrophages and Monocytes | 0.816 | ND |
| Germ Cell-Sertoli Cell Junction Signaling | ND | ND |
| Glucocorticoid Receptor Signaling | ND | ND |
| Gluconeogenesis I | -1.89 | ND |
| Glycerol Degradation I | ND | ND |
| Glycerol-3-phosphate Shuttle | ND | ND |
| Glycolysis I | -1.342 | ND |
| GP6 Signaling Pathway | -0.707 | -1.342 |
| Hepatic Fibrosis / Hepatic Stellate Cell Activation | ND | ND |
| Hepatic Fibrosis Signaling Pathway | 0 | -1.342 |
| HER-2 Signaling in Breast Cancer | 2.111 | 0 |
| IL-8 Signaling | 0 | -2.236 |
| ILK Signaling | 1.387 | 0.447 |
| Integrin Signaling | 2.324 | 0 |
| Intrinsic Prothrombin Activation Pathway | -1.342 | ND |
| Leukocyte Extravasation Signaling | 0.333 | -0.447 |
| Melatonin Degradation III | ND | ND |
| Mitochondrial Dysfunction | ND | ND |
| Natural Killer Cell Signaling | 0.905 | 1 |
| Oxidative Phosphorylation | -6.782 | -2.449 |
| Paxillin Signaling | 2.646 | ND |
| Rapoport-Luebering Glycolytic Shunt | ND | ND |
| Regulation of Actin-based Motility by Rho | 0.816 | ND |
| Remodeling of Epithelial Adherens Junctions | ND | ND |
| RhoGDI Signaling | -1.134 | ND |
| Role of IL-17A in Psoriasis | ND | ND |
| Semaphorin Neuronal Repulsive Signaling Pathway | -0.905 | ND |
| Signaling by Rho Family GTPases | 2.138 | ND |
| Sirtuin Signaling Pathway | 3.904 | 0.707 |
| Synaptogenesis Signaling Pathway | 2.673 | 1.633 |
| Tight Junction Signaling | ND | ND |

ND; directionality not determined.

**Supplemental Table 3.** Directionality of Pathways (based on z-score) Altered in the Phosphoproteomes of Both Sarcomere-Positive HCM and Genotype-Negative HCM Compared with Controls.

| Ingenuity Canonical Pathways | Sarcomere-Positive HCM versus Control | Genotype-Negative HCM versus Control |
| --- | --- | --- |
| 14-3-3-mediated Signaling | 0 | -1.667 |
| 3-phosphoinositide Biosynthesis | -1.604 | -1 |
| 3-phosphoinositide Degradation | -0.905 | 0 |
| Actin Cytoskeleton Signaling | 1.091 | -1.155 |
| Acute Myeloid Leukemia Signaling | -0.333 | -1.414 |
| Adrenomedullin signaling pathway | -0.688 | -2.357 |
| Aldosterone Signaling in Epithelial Cells | -0.832 | -1.667 |
| AMPK Signaling | -0.535 | -1.732 |
| Angiopoietin Signaling | ND | ND |
| Apelin Cardiomyocyte Signaling Pathway | -0.535 | -1.89 |
| Autophagy | -0.471 | 0.277 |
| Axonal Guidance Signaling | ND | ND |
| BAG2 Signaling Pathway | 0.378 | 0 |
| BEX2 Signaling Pathway | 0.707 | -0.447 |
| Cardiac Hypertrophy Signaling | -1.528 | -3.051 |
| Cardiac Hypertrophy Signaling (Enhanced) | -0.87 | -2.357 |
| CCR3 Signaling in Eosinophils | -0.816 | -1.89 |
| CDK5 Signaling | -1.414 | -0.447 |
| Ceramide Signaling | -1 | 0.816 |
| Cholecystokinin/Gastrin-mediated Signaling | -0.632 | -1.89 |
| Chronic Myeloid Leukemia Signaling | ND | ND |
| CNTF Signaling | -0.816 | -2 |
| Colorectal Cancer Metastasis Signaling | -0.905 | -2.333 |
| Coronavirus Replication Pathway | -0.816 | -1 |
| Cyclins and Cell Cycle Regulation | 1.633 | 1.342 |
| D-myo-inositol (1,4,5)-Trisphosphate Biosynthesis | 1 | ND |
| D-myo-inositol (1,4,5,6)-Tetrakisphosphate Biosynthesis | -1.265 | 0 |
| D-myo-inositol (3,4,5,6)-tetrakisphosphate Biosynthesis | -1.265 | 0 |
| D-myo-inositol-5-phosphate Metabolism | -0.577 | -0.333 |
| Docosahexaenoic Acid (DHA) Signaling | ND | ND |
| Dopamine-DARPP32 Feedback in cAMP Signaling | 0.277 | -2.121 |
| EGF Signaling | -0.707 | -2.828 |
| EIF2 Signaling | -1.155 | -2.333 |
| Endocannabinoid Cancer Inhibition Pathway | -0.832 | 0.302 |
| Endocannabinoid Developing Neuron Pathway | 0.333 | -1.633 |
| Endometrial Cancer Signaling | -0.707 | -2.449 |
| Endothelin-1 Signaling | -0.832 | -2.309 |
| eNOS Signaling | -0.905 | -2.121 |
| Ephrin A Signaling | ND | ND |
| ERB2-ERBB3 Signaling | -0.302 | -2.333 |
| ERBB Signaling | -0.905 | -2.53 |
| ERBB4 Signaling | -1.414 | -2.646 |
| ERK/MAPK Signaling | 1.528 | -1.147 |
| Erythropoietin Signaling Pathway | -1.155 | -3.162 |
| Estrogen Receptor Signaling | -1.521 | -2.4 |
| Factors Promoting Cardiogenesis in Vertebrates | -0.905 | -1.414 |
| Fc Epsilon RI Signaling | -1.667 | -3 |
| FcγRIIB Signaling in B Lymphocytes | -1 | -2.236 |
| FGF Signaling | -0.577 | -3 |
| FLT3 Signaling in Hematopoietic Progenitor Cells | -0.333 | -2.333 |
| G Beta Gamma Signaling | 0 | -1.633 |
| GADD45 Signaling | 0.447 | 0 |
| Gap Junction Signaling | ND | ND |
| GDNF Family Ligand-Receptor Interactions | -1.667 | -3 |
| Germ Cell-Sertoli Cell Junction Signaling | ND | ND |
| Glioblastoma Multiforme Signaling | -1.291 | -2.53 |
| Glioma Signaling | -1.414 | -2.646 |
| GM-CSF Signaling | -1.134 | -2.449 |
| GP6 Signaling Pathway | -0.333 | -1.667 |
| G-Protein Coupled Receptor Signaling | 1.441 | -1.706 |
| Gαi Signaling | -0.302 | -0.816 |
| Gαq Signaling | -0.577 | -1.414 |
| Hepatic Fibrosis Signaling Pathway | -0.756 | -1.807 |
| HER-2 Signaling in Breast Cancer | 0 | -2.673 |
| HGF Signaling | -0.632 | -2.121 |
| HIF1α Signaling | -1.291 | -1.667 |
| Huntington's Disease Signaling | -0.632 | -2.333 |
| ID1 Signaling Pathway | 0.655 | -0.302 |
| IGF-1 Signaling | -0.577 | -2.53 |
| IL-2 Signaling | -1.414 | -2.646 |
| IL-3 Signaling | -0.707 | -1.89 |
| IL-6 Signaling | -1.508 | -3 |
| IL-7 Signaling Pathway | -0.333 | -2.309 |
| ILK Signaling | 1.342 | 0.632 |
| Inhibition of ARE-Mediated mRNA Degradation Pathway | 0.277 | 0 |
| Insulin Receptor Signaling | -1 | -1.941 |
| Insulin Secretion Signaling Pathway | 0.775 | -2.673 |
| Integrin Signaling | 0.894 | -1.732 |
| JAK/STAT Signaling | -1.134 | -2.449 |
| Leptin Signaling in Obesity | -0.447 | ND |
| LPS-stimulated MAPK Signaling | -0.378 | -1.342 |
| Melanocyte Development and Pigmentation Signaling | -1 | -2.828 |
| Melanoma Signaling | -0.447 | -2 |
| Melatonin Signaling | 0.632 | -0.447 |
| Molecular Mechanisms of Cancer | ND | ND |
| Mouse Embryonic Stem Cell Pluripotency | -1.414 | -2.646 |
| MSP-RON Signaling In Cancer Cells Pathway | 0.577 | -1.89 |
| mTOR Signaling | 0 | -0.378 |
| NAD Signaling Pathway | -2.53 | -2.828 |
| Natural Killer Cell Signaling | -0.5 | -1.941 |
| Neuregulin Signaling | -0.632 | -2.121 |
| Neuropathic Pain Signaling In Dorsal Horn Neurons | 0 | -1.89 |
| Neurotrophin/TRK Signaling | -0.632 | -2.121 |
| NGF Signaling | -1.265 | -2.646 |
| Non-Small Cell Lung Cancer Signaling | 0.302 | -2.121 |
| NRF2-mediated Oxidative Stress Response | 0.816 | ND |
| Oxytocin Signaling Pathway | 0.962 | -2.668 |
| P2Y Purigenic Receptor Signaling Pathway | -0.277 | -1.89 |
| p70S6K Signaling | -0.471 | -1.941 |
| PAK Signaling | -1.387 | -2.333 |
| Pancreatic Adenocarcinoma Signaling | 0 | -2 |
| Paxillin Signaling | 0.302 | -1.897 |
| PD-1, PD-L1 cancer immunotherapy pathway | 0.378 | 1.633 |
| PDGF Signaling | -1.134 | -2.121 |
| Phagosome Formation | 0.686 | -2.4 |
| PI3K Signaling in B Lymphocytes | 0.577 | -1.134 |
| PI3K/AKT Signaling | 1.155 | -0.905 |
| PPARα/RXRα Activation | 0.333 | 0 |
| Production of Nitric Oxide and Reactive Oxygen Species in Macrophages | -0.577 | -1.414 |
| Prolactin Signaling | -1.414 | -2.646 |
| Prostate Cancer Signaling | ND | ND |
| Protein Kinase A Signaling | -0.18 | -0.577 |
| PTEN Signaling | 1.155 | 1.508 |
| Pulmonary Healing Signaling Pathway | -0.577 | 0.333 |
| RAC Signaling | -0.333 | -1.134 |
| RANK Signaling in Osteoclasts | -0.707 | -1.89 |
| Regulation of eIF4 and p70S6K Signaling | -0.632 | -1.897 |
| Regulation Of The Epithelial Mesenchymal Transition By Growth Factors Pathway | 0 | -1.732 |
| Regulation of the Epithelial-Mesenchymal Transition Pathway | ND | ND |
| Renal Cell Carcinoma Signaling | -0.707 | -1.633 |
| Renin-Angiotensin Signaling | -1.604 | -2.887 |
| RHOA Signaling | 1.069 | 1.89 |
| Role of NANOG in Mammalian Embryonic Stem Cell Pluripotency | -1.414 | -2.646 |
| Role of NFAT in Cardiac Hypertrophy | 0 | -2.111 |
| Role of p14/p19ARF in Tumor Suppression | ND | ND |
| Role of PI3K/AKT Signaling in the Pathogenesis of Influenza | -0.447 | -2 |
| Role of Tissue Factor in Cancer | ND | ND |
| Semaphorin Neuronal Repulsive Signaling Pathway | 0.243 | 1.604 |
| Senescence Pathway | 0.218 | -1.291 |
| Signaling by Rho Family GTPases | 0.894 | 0 |
| Small Cell Lung Cancer Signaling | 0 | ND |
| Sperm Motility | -0.302 | -1.89 |
| Sphingosine-1-phosphate Signaling | -0.632 | -1.89 |
| Superpathway of Inositol Phosphate Compounds | -1 | -1.155 |
| Synaptogenesis Signaling Pathway | 0.426 | -2.236 |
| Telomerase Signaling | -0.333 | -1.667 |
| TGF-β Signaling | -0.378 | -1.342 |
| Thrombin Signaling | -0.258 | -2.333 |
| Thrombopoietin Signaling | -1.89 | -2.646 |
| Thyroid Cancer Signaling | -0.816 | -2.449 |
| TR/RXR Activation | ND | ND |
| Unfolded protein response | ND | ND |
| UVA-Induced MAPK Signaling | -0.378 | -1.633 |
| UVB-Induced MAPK Signaling | -0.447 | -1.342 |
| VEGF Family Ligand-Receptor Interactions | -1.414 | -2.828 |
| VEGF Signaling | -0.905 | -2.53 |
| WNT/Ca+ pathway | 1.134 | 0.447 |
| Xenobiotic Metabolism General Signaling Pathway | -0.632 | -1.414 |

ND; directionality not determined.

**Supplemental Figure Captions**

**Supplemental Figure 1.** Additional Information for Comparison Between HCM_Sarc_ and HCM_Neg_. A) Principal component analysis (PCA) plot shows no separation between the proteomes of HCM_Sarc_ and HCM_Neg_. B) Volcano plot of proteomics data comparing HCM_Sarc_ and HCM_Neg_ using a threshold of adjusted (adj) p-value ≥ 0.05 and log2 fold change (log2fc) ≥ |0.5|. C) Principal component analysis (PCA) plot shows some separation between phosphoproteome of HCM_Sarc_ and HCM_Neg_. D) Volcano plot of phosphoproteomics data comparing HCM_Sarc_ and HCM_Neg_ using a threshold of adjusted (adj) p-value ≥ 0.05 and log2 fold change (log2fc) ≥ |0.5|.

**Supplemental Figure 2.** Additional Information for Comparison Between HCM_Sarc_ and Controls. A) Principal component analysis (PCA) plot showing separation between the proteomes of HCM_Sarc_ and control samples. B) Volcano plot of proteomics data comparing HCM_Sarc_ with controls using a threshold of adjusted (adj) p-value ≥ 0.05 and log2 fold change (log2fc) ≥ |0.5|. C) Principal component analysis (PCA) plot showing separation between phosphoproteome of HCM_Sarc_ and control samples. D) Volcano plot of phosphoproteomics data comparing HCM_Sarc_ and controls using a threshold of adjusted (adj) p-value ≥ 0.05 and log2 fold change (log2fc) ≥ |0.5|.

**Supplemental Figure 3.** Comparison Between HCM_Sarc_ and Controls. A) Most up- and down-regulated Gene Ontology (GO) biological processes in proteome using gene set enrichment analysis (GSEA). B) Most altered Gene Ontology (GO) biological processes in phosphoproteome using gene set enrichment analysis (GSEA). C) Most statistically altered pathways (largest -log [BH p-value]) in proteome. D) Top up- and down-regulated (z-score ≥ |1|) pathways in proteome. E) Most statistically altered phosphorylation (largest -log [BH p-value]) of pathways. F) Top activated and inactivated pathways based on phosphorylation (z-score ≥ |1|).

**Supplemental Figure 4.** Additional Information for HCM_Neg_ and Controls. A) Principal component analysis (PCA) plot showing separation between proteome of HCM_Neg_ and control samples. B) Volcano plot of proteomics data comparing HCM_Neg_ with controls using a threshold of adjusted (adj) p-value ≥ 0.05 and log2 fold change (log2fc) ≥ |0.5|. C) Principal component analysis (PCA) plot showing separation between phosphoproteome of HCM_Neg_ and control samples. D) Volcano plot of phosphoproteomics data comparing HCM_Neg_ and controls using a threshold of adjusted (adj) p-value ≥ 0.05 and log2 fold change (log2fc) ≥ |0.5|.

**Supplemental Figure 5.** Comparison of Proteome and Phosphoproteome of HCM_Neg_ and Controls. A) Most up- and down-regulated Gene Ontology (GO) biological processes in proteome using gene set enrichment analysis (GSEA). B) Most statistically altered pathways (largest -log [BH p-value]) in proteome. C) Top up- and down-regulated (z-score ≥ |1|) pathways in proteome. D) Most statistically altered phosphorylation (largest -log [BH p-value]) of pathways. E) Top activated and inactivated pathways based on phosphorylation (z-score ≥ |1|).

* Negative Regulation of Transmembrane Receptor Protein Serine Threonine Kinase Signaling Pathway was shortened.

**Supplemental Figure 6.** Network Analysis of Differentially Expressed Proteins Unique to HCM_Sarc_. A) Protein-protein interaction network of proteins using STRINGS database. B) Top Gene ontology (GO) biological processes enriched in protein list.

**Supplemental Figure 7.** Venn Diagrams for Gene Ontology Biological Processes (GOBPs) Altered Betweeen HCM_Sarc_ and HCM_Neg_. A) GOBPs altered in the proteome. B) GOBPs altered in phosphoproteome.
